# Quantitative Modeling of Microbial Population Responses to Chronic Irradiation Combined with Other Stressors

**DOI:** 10.1371/journal.pone.0147696

**Published:** 2016-01-25

**Authors:** Igor Shuryak, Ekaterina Dadachova

**Affiliations:** 1 Center for Radiological Research, Columbia University, New York, NY, United States of America; 2 Department of Radiology, Albert Einstein College of Medicine, Bronx, New York, United States of America; 3 Department of Microbiology and Immunology, Albert Einstein College of Medicine, Bronx, New York, United States of America; University of South Carolina, UNITED STATES

## Abstract

Microbial population responses to combined effects of chronic irradiation and other stressors (chemical contaminants, other sub-optimal conditions) are important for ecosystem functioning and bioremediation in radionuclide-contaminated areas. Quantitative mathematical modeling can improve our understanding of these phenomena. To identify general patterns of microbial responses to multiple stressors in radioactive environments, we analyzed three data sets on: (1) bacteria isolated from soil contaminated by nuclear waste at the Hanford site (USA); (2) fungi isolated from the Chernobyl nuclear-power plant (Ukraine) buildings after the accident; (3) yeast subjected to continuous γ-irradiation in the laboratory, where radiation dose rate and cell removal rate were independently varied. We applied generalized linear mixed-effects models to describe the first two data sets, whereas the third data set was amenable to mechanistic modeling using differential equations. Machine learning and information-theoretic approaches were used to select the best-supported formalism(s) among biologically-plausible alternatives. Our analysis suggests the following: (1) Both radionuclides and co-occurring chemical contaminants (e.g. NO_2_) are important for explaining microbial responses to radioactive contamination. (2) Radionuclides may produce non-monotonic dose responses: stimulation of microbial growth at low concentrations vs. inhibition at higher ones. (3) The extinction-defining critical radiation dose rate is dramatically lowered by additional stressors. (4) Reproduction suppression by radiation can be more important for determining the critical dose rate, than radiation-induced cell mortality. In conclusion, the modeling approaches used here on three diverse data sets provide insight into explaining and predicting multi-stressor effects on microbial communities: (1) the most severe effects (e.g. extinction) on microbial populations may occur when unfavorable environmental conditions (e.g. fluctuations of temperature and/or nutrient levels) coincide with radioactive contamination; (2) an organism’s radioresistance and bioremediation efficiency in rich laboratory media may be insufficient to carry out radionuclide bioremediation in the field—robustness against multiple stressors is needed.

## Introduction

Understanding radionuclide effects on non-human biota is important for environmental protection and bioremediation after accidental (e.g. from nuclear power plants or waste storage facilities) or malicious (“dirty bomb” attacks) radionuclide releases [1–7]. Microbial population responses in radionuclide-contaminated environments can differ from those measured experimentally because the biological effects of radionuclides are modulated by other stressors (e.g. chemical contaminants, starvation) which the target organisms are subjected to [8].

The objective of this paper is to use quantitative mathematical modeling to identify general patterns of microbial responses to radioactive contamination. Understanding and predicting such patterns can guide research on environmental protection and bioremediation after radionuclide releases [5, 6, 8]. In particular, study results could be useful in identifying which microbial taxa are most sensitive to radioactive pollution and, conversely, which are most robust and could, therefore, be considered as bioremediation agents [7, 9].

Unfortunately, data on multi-stressor combinations involving chronic ionizing radiation exposure are limited [2, 10, 11] and extrapolation from acute to chronic irradiation can be unreliable [8, 12]. We searched the literature for published data sets on microbial populations subjected to chronic irradiation, which met the following conditions: radiation had to be combined with other stressors (e.g. chemical toxicants, oligotrophic conditions, and/or high cell removal rates), and the publications had to contain enough information to conduct a thorough mathematical/statistical analysis. The search was not intended to be exhaustive. Instead, we sought to use data sets on different microbial groups (e.g. bacteria and fungi) and exposure conditions (e.g. field and laboratory), so that integrating the analysis results from all these data sets would help to form a general picture of how microbial communities respond to chronic irradiation combined with other stressors.

The first data set was generated by bacteriological analysis of vadose sediments located under a high-level radioactive waste storage tank at the Hanford site, near Richland, Washington [13]. We selected it as an informative example of bacterial population responses, under field conditions, to combined stresses from radiation, chemical toxicants, and unfavorable environmental factors (limited soil water content, oligotrophic conditions, high temperature). Sixteen samples were collected at various depths beneath the tank 40–50 years after it leaked, releasing radionuclides and chemical contaminants [13]. Unfortunately, only one contaminated site was sampled, but we believe that it is sufficiently representative to support a modeling analysis. Two control samples were obtained from a nearby unpolluted location. Temperature, pH, water content, conductivity, concentrations of ^137^Cs, ^99^Tc, Cr, NO_3_ and NO_2_, and the identities of detected bacterial taxa were recorded for each sample [13]. ^137^Cs is a high energy beta and gamma emitter with a relatively long physical half-life of 30 years. Its salts are easily soluble in water and it is present in the solution as a monovalent cation which can pollute the ground waters and in vivo mimics potassium behavior. ^99^Tc is a weak beta emitter with a long physical half-life of 10^5^ years, it primarily exists in its two most stable and chemically inert forms - ^99^TcO_2_ oxide and ^99^Tc-perrhenate. Cr (in its chromate form), NO_3_ and NO_2_ are powerful chemical oxidants. The radiation dose rate from ^137^Cs (the predominant radionuclide) reached 24–370 mGy/h (using mean energy/decay coefficients from reference [14]) in the three most radioactive samples from which viable bacteria were isolated.

**Fig 1:**
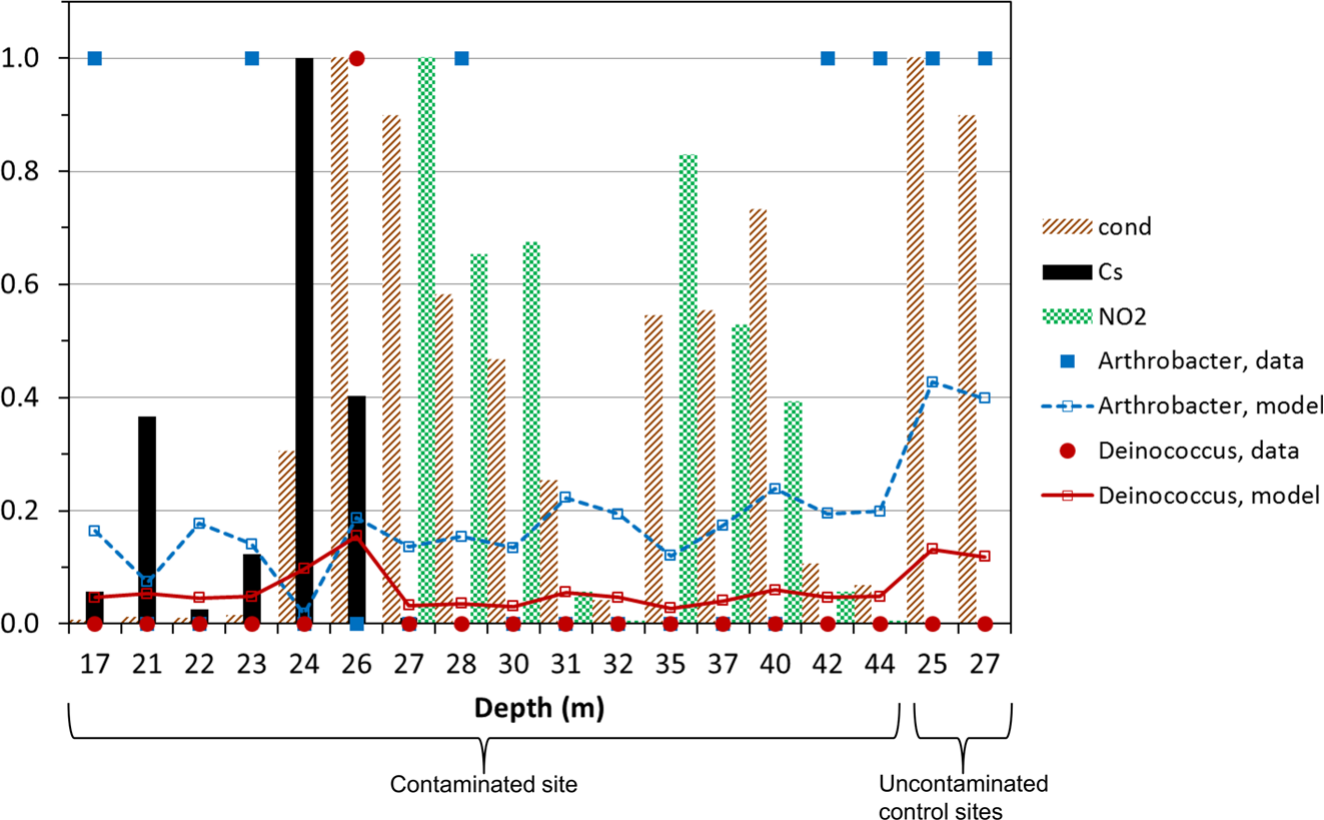
Comparison of best-fitting mixed-effect model (Table 1, S2 Appendix) predictions with the data on bacteria at the Hanford nuclear waste site (data set one, reference [13], S1 Appendix). The y-axis represents: (1) the values of various stressors (bars), normalized (for convenience of presentation) by the maximum measured value within the data set; and (2) presence/absence data (points and lines) for bacterial genera. Two genera were selected in this illustration: *Arthrobacter*, which is common in vadose sediments at Hanford and sensitive to radiation, and *Deinococcus*, which was isolated only from one sample with high ^137^Cs levels (indicated by the arrow) and is known to be radioresistant [13]. Closed symbols represent observations, and open symbols and lines represent best-fitting mixed-effect model predictions. Cond = conductivity.

**Fig 2:**
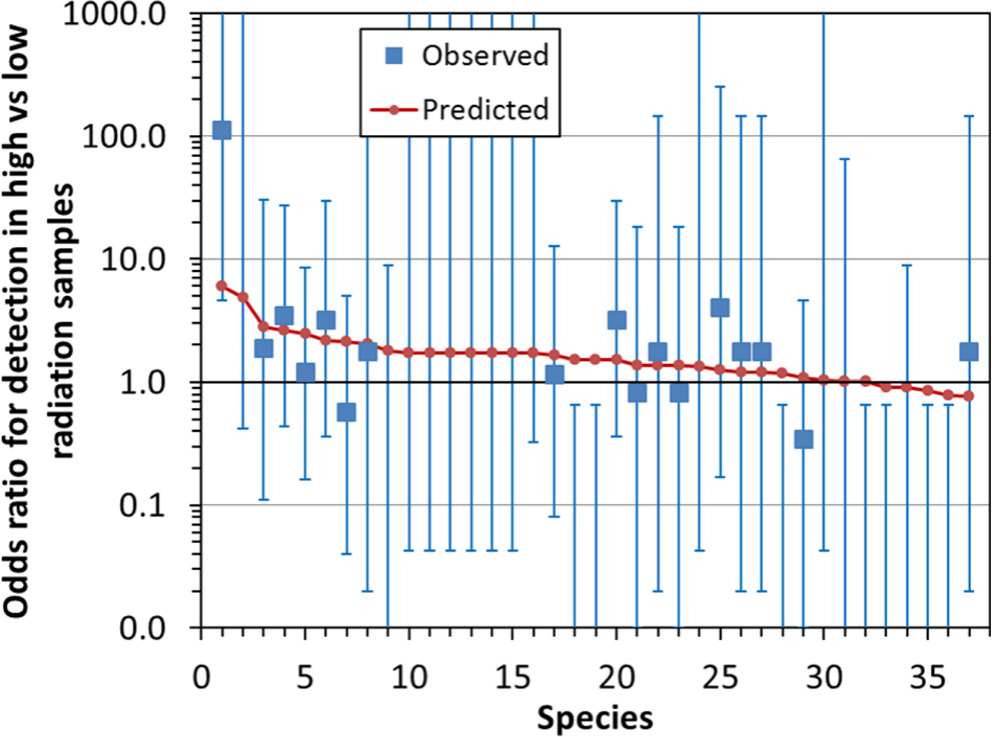
Comparison of observed (squares) and best-fit model predicted (circles) odds ratios for detection of fungal species in locations with high vs. low dose rates within the Chernobyl reactor buildings (data set two, reference [18], S1 Appendix). Error bars indicate 95% confidence intervals (CIs) calculated by Fisher’s exact test with Type I error threshold of 0.05. The predictions are points, and they are connected by a line just for convenience to guide the eye. No points are shown when odds ratio estimates equaled zero or infinity, but CI bounds are shown in these cases. The species are: 1 *= Penicillium hirsutum*, 2 = *Cladosporium sphaerospermum*, 3 = *Aspergillus niger*, 4 = *Aureobasidium versicolor*, 5 = *Alternaria alternata*, 6 = *Aureobasidium pollulans*, 7 = *Acremonium strictum*, 8 = *Aspergillus fumigatus*, 9 = *Penicillium ingelheimense*, 10 = *Aspergillus flavus*, 11 = *Aspergillus fresenii*, 12 = *Aspergillus ochraceus*, 13 = *Aspergillus ustus*, 14 = *Paecilomyces variotii*, 15 = *Penicillium citrinum*, 16 = *Phialophora melinii*, 17 = *Chaetomium globosum*, 18 = *Penicillium chrysogenum*, 19 = *Penicillium hordei*, 20 = *Cladosporium herbarum*, 21 = *Botrytis cinerea*, 22 = *Doratomyces stemonitis*, 23 = *Fusarium solani*, 24 = *Geotrichum candidum*, 25 = *Ulocladium (white sterile mycelium)*, 26 = *Fusarium oxysporum*, 27 = *Stachybotrys chartarum*, 28 = *Geotrichum sp*., 29 = *Cladosporium cladosporioides*, 30 = *Beauveria bassiana*, 31 = *Chrysosporium pannorum*, 32 = *Mucor plumbeus*, 33 = *Fusarium merismoides*, 34 = *Ulocladium (orange sterile mycelium)*, 35 = *Sydowia polyspora*, 36 = *Ulocladium botrytis*, 37 = *Cladosporium sp*. They are arranged on the x-axis in rank order of predicted odds ratios.

We used this information (presented mainly in Tables 1 and 3 and in Figs. 1 and 2 of reference [13], and combined in our S1 Appendix) to compile a data set which consisted of binary (1 = presence, 0 = absence) values for each taxon in each soil sample. The bacteria were isolated by cultivation which focused on aerobic chemoheterotrophic bacteria, but also included enrichments for select physiological groups of anaerobic bacteria [13]. Some taxa were identified to the species level, whereas for others only a broad taxonomic category was assigned. We chose genus as the most representative category. The most frequently detected genus was *Arthrobacter*, which is often common in soil [15], but many other genera, e.g. *Deinococcus* [16, 17], were also identified [13].

Using this data set we sought to evaluate whether the toxicity of nuclear waste to soil bacteria was driven mainly by radionuclides (^137^Cs, ^99^Tc), or whether chemical toxicants (Cr, NO_3_, NO_2_) were also important. In addition, we sought to evaluate how variability of responses to the various toxicants among different bacterial taxa could affect community composition in contaminated soil: for example, would the most radioresistant taxa gain a competitive advantage over radiosensitive ones.

The second data set was generated by mycological analysis of 24 samples collected within the Chernobyl nuclear power plant buildings 11–12 years after the accident [18]. It is an example of how fungi (37 species) are affected by severe radioactive contamination under oligotrophic conditions. Samples were collected by sterile cotton swabs and stamped onto solid agar growth medium. The samples were separated into two groups based on radioactive contamination levels: low dose rate (1.5–25 mR/h of γrays, 15 samples) and high dose rate (40–220 mR/h, 9 samples). The γ-ray measurements were only a surrogate for the higher total dose rate, mainly generated by α- and β-emitting radionuclides [18]. The proportions of samples at low and high dose rate locations in which each fungal species was detected are presented in Table 1 of reference [18] and in our S1 Appendix.

Using this data set, we sought to evaluate how the approximately 10-fold difference in radiation dose rate within the reactor buildings affected fungal community composition. Specifically, we intended to identify which fungal taxa are most robust under chronic irradiation and oligotrophic conditions, and would therefore be potentially useful as radioactive waste bioremediators.

The third data set was produced by continuous ^60^Co γ-irradiation of diploid yeast (*Saccharomyces cerevisiae*, strain 211) in a laboratory chemostat for multiple generations [19]. This data set (presented in Tables 1–2 of reference [19] and in our S1 Appendix) qualitatively differs from the previous two because it was generated under laboratory, rather than field, conditions. Consequently, the specifics of culturing in the laboratory may exert their own effect, different from the effects of radiation. However, we selected this data set because laboratory studies have the advantage that the investigated stressors are more easily controlled and quantified, than in field studies, and there is less potential for confounding by unknown factors.

In this third data set we sought to investigate how the critical radiation dose rate, above which population extinction occurs, is modulated by an experimentally-controlled adverse factor–the cell removal rate. The liquid culture medium flowed through the chemostat at a constant rate (the dilution rate) fixed by the experimenters. The dilution rate thus represented the cell removal rate, which acted as an additional stressor–an example of unfavorable conditions which increase mortality in the population. Since dilution rate and dose rate were varied independently, the effects of each stressor could be readily assessed and disentangled.

The three data sets were purposely selected to be diverse and to include a wide range of microbial taxa, exposure conditions, and stressors. Our goal was to integrate the analysis results from each data set by formulating general conclusions with potentially broad applicability to environmental protection and radioactive waste bioremediation. We used descriptive and (whenever possible) mechanistic mathematical modeling, which involved machine learning and/or information theoretic model selection, to analyze the three data sets and provide insight into environmental effects of radionuclides combined with other stressors [11, 20–22].

## Materials and Methods

### Data Set One: Bacteria at the Hanford Nuclear Waste Site

We modeled the probability of detecting a given bacterial taxon in each soil sample using logistic regression, where the radionuclides, chemical toxicants and measured soil properties (e.g. conductivity) were the predictor variables. To account for potential differences in how strongly different taxa respond to particular stressors (e.g. ^137^Cs), we used generalized linear mixed-effects models (GLMMs), which extend logistic regression to include both fixed and random effects. Using matrix notation, GLMM structure is summarized as follows, where logit(*x*) = 1/(1+exp(*x*)):

logit(o)=α×V+β×φ+ε (1)

Here, ***o*** is a vector of outcome variables: predicted probabilities of isolating a given genus in a given sample. ***V*** is a matrix of predictor variables: sample-specific concentrations of radionuclides (^137^Cs, ^99^Tc) and chemical contaminants (Cr, NO_3_ and NO_2_), and environmental conditions (depth below the soil surface, temperature, pH, water content, conductivity). **α** is a vector of fixed-effect regression coefficients. ***φ***is a matrix of random effects, which model differences between genera in baseline abundance (intercepts) and responses (slopes) to variables of interest. These random effects represent genus-specific deviations from the corresponding fixed effect (e.g. variations in sensitivity to radiation from ^137^Cs). They are normally distributed with a mean of zero and an adjustable variance. **β** is a vector of random effect coefficients, and **ε** is a vector of binomially-distributed errors.

Because the measured radionuclides (^137^Cs, ^99^Tc) and chemical contaminants (Cr, NO_3_, NO_2_) were released from the same source (the ruptured nuclear waste tank), their distributions in soil were correlated (e.g. the correlation coefficient of Cr with NO_2_ was 0.83). Consequently, several different combinations of variables could potentially describe the outcome equally well, and multi-collinearity was a potentially important problem. To identify those variables with the strongest main effects, we employed a customized approach (called, for convenience, the “filter procedure”). This procedure, described in detail in the S2 Appendix, used machine learning and information theoretic methods implemented in R software (version 3.2.2).

### Data Set Two: Fungi in Chernobyl Reactor Buildings

We modeled the probability for detection of each fungal species in each sample using the same GLMM approach as described above. Since only one environmental variable–radiation dose rate–was reported, there was no need for the filter procedure. Oligotrophic conditions, which probably had an important impact on fungal growth, were not described in quantitative terms and therefore could not be modeled. Unfortunately, there was no information provided in reference [18] about the spatial locations of samples and about randomness of their collection, but we made efforts to mitigate this by assessing the effects of potential correlations between samples as described in the S3 Appendix.

We used a GLMM with a fixed effect of radiation (coded as a binary variable: 0 = low and 1 = high dose rate) and random effects (intercepts and slopes) for two taxonomic variables: order and species. The sum of fixed and random effects for each species produced the net model coefficient for radiation effect on the given species. This coefficient was converted to the odds ratio for detection of a given species in high vs. low radiation locations. This predicted odds ratio was compared to the observed odds ratio (with 95% confidence intervals estimated by Fisher’s exact test with Type I error threshold of 0.05). Absolute goodness of fit (GOF) for the GLMMs was assessed by marginal R^2^, which represents the variance explained by fixed effects, and conditional R^2^, which represents variance explained by both fixed and random effects (i.e. by the entire model) [23, 24].

We also investigated whether the lethal dose of acute irradiation, taken from reference [25], is associated with detection of a given taxon in the reactor buildings–e.g., if more radioresistant fungi are more likely to have been found in these heavily contaminated structures. We created a data set consisting only of those species for which the lethal dose of acute irradiation was reported in reference [25], and manually added this lethal dose (in kGy), and the interaction of the lethal dose with the binary radiation dose rate category, as additional predictors in the GLMM.

Since the GLMM analysis does not explicitly account for inter-specific interactions, we calculated the bias-corrected Chao2 species richness estimator [26, 27] for the low and high dose rate locations separately. Significant differences between the values of this estimator could result from phenomena such as elimination of radiosensitive species and selection of radioresistant ones in the most contaminated areas [28].

### Data Set Three: Continuously Irradiated Yeast

The endpoints of interest in this data set were: (1) the equilibrium cell concentration, i.e. the number of cells per ml of growth medium when a steady-state of the system has been achieved and no further changes were detectable; and (2) the critical dose rate above which population extinction occurred (i.e. no steady-state could be achieved). To model these endpoints, we used mechanistic approaches, which provides important advantages over purely descriptive ones [29, 30]. We were able to do so for this data set because only one taxon and only two predictor variables (radiation and chemostat dilution) were involved. Although the presence of only one taxon is a limitation because interactions (e.g. competition) between taxa cannot be studied, the simplicity of this system also provides an advantage because stressor effects on the single taxon can be examined without confounding. Twenty distinct formalisms (M_1_-M_20_), which modeled the rate of change of the cell concentration over time using differential equations, were tested. These equations contained terms representing cell proliferation, death, intra specific competition, and the effects of radiation and dilution rate. S4 Appendix describes the procedure applied to a fully worked example using M_1_.

## Results

### Data Set One: Bacteria at the Hanford Nuclear Waste Site

This data set provides detailed information on how a bacterial community, which exists under harsh conditions (in sub-surface sediments with low water content and nutrient concentrations), responded to severe contamination with radionuclides and chemical toxicants [13]. There was no evidence that the composition of bacterial taxa detected in any two samples was correlated with the depth of soil separating the samples: the Mantel test for spatial auto-correlation using 9999 random permutations produced a correlation of only -0.02 with a p-value of 0.81. Consequently, we proceeded to treat the samples collected at different depths as statistically independent.

Eight potential predictor variables for the probability to detect each bacterial taxon (water content, conductivity, temperature, ^137^Cs, ^99^Tc, Cr, NO_3_ and NO_2_) were identified as having important main effects using machine learning methods implemented as part of the filter procedure described in S2 Appendix. In contrast, the remaining two variables (depth below the soil surface and pH) demonstrated low importance for describing the data and were excluded from further analysis. The low importance of pH was somewhat surprising because the effect of pH on bacterial diversity is well known. Therefore, we checked this finding by manually including pH in the logistic model. The regression coefficient was not statistically significant: 0.246 (SE: 0.433, P = 0.57), confirming that this variable was unimportant. Consequently, pH was not used for further analysis.

The variance inflation factors (VIF) were high (>5) for temperature, Cr and NO_3_. Consequently, these three variables were removed from the model to alleviate multi-collinearity. The resulting confidence set of fixed-effect models contained only water content, conductivity, ^137^Cs, ^99^Tc, and NO_2_, with VIFs of 1.12, 1.09, 1.10, 1.79, and 1.79, respectively. Multi-model inference (MMI, described in S2 Appendix) suggested that all of these variables exhibited comparable importance (Table 1). For example, the ratio of importances for the most important variable (137Cs) to the least important (water content) was only 0.99/0.57 = 1.7.

**Table 1:** Summary of quantitative models used to describe the data on bacteria at the Hanford nuclear waste site (data set one, reference [13], S1 Appendix). Cond = conductivity. SE = standard errors of the model coefficients. NA = non-applicable. Details are provided in the main text and in S2 Appendix.

Variable (units)	MMI using confidence set of fixed-effect models				Best-fitting mixed-effect model			Standard deviation of random effect
	Impor- tance	Coeffi- cient	SE	p-value	Coeffi- cient	SE	p-value	
Water content (%)	0.57	-0.0301	0.037	0.418				
Cond (mS/cm)	0.85	0.0167	0.010	0.107	0.0215	0.008	0.008	NA
^137^Cs (μCi/g)	0.99	-0.0763	0.033	0.023				0.0247
^99^Tc (μCi/L)	0.62	-0.0046	0.005	0.363				
NO_2_ (mg/L)	0.70	-0.0118	0.011	0.273	-0.0160	0.007	0.021	NA
Intercept	NA	–1.9122	0.505	1.6×10^−4^	-2.8369	0.337	2×10^−16^	0.6181

These results suggest that conductivity had a marginally positive effect on bacteria (Table 1), which may reflect a correlation between conductivity and nutrient concentrations. Water content had a non-significant negative effect. The radionuclides ^137^Cs and ^99^Tc had negative effects, which reached statistical significance for ^137^Cs (Table 1). NO_2_ also had a non-significant negative effect (Table 1), which may reflect its own toxicity as well as correlations with other chemical toxicants which were not retained in the model as independent predictors.

The best-supported fixed effect model contained only conductivity, ^137^Cs and NO_2_ as predictors. The VIFs for these variables were 1.04, 1.04 and 1.08, respectively. Eight observations (out of 460) exceeded the Cook's distance threshold for outlier data points (defined in S2 Appendix), but their effects were minor: the comparison of robust vs regular logistic regression demonstrated that model coefficient values hardly changed and none of them changed sign. There was no evidence of overdispersion: logistic regression with a quasibinomial error distribution produced a dispersion parameter of 0.95, and the p-value of the X^2^ test comparing the fits of quasibinomial and binomial regressions was 0.79. There was also no evidence of autocorrelation of residuals: the Durbin Watson test p-value was 0.49.

Even though the best-supported fixed-effect model assumes that the responses of all taxa to a given predictor are identical, it achieved fair GOF (R^2^ = 0.512) and predictive accuracy: area under the Receiver Operating Characteristic (ROC) curve [31] and adjusted error rate from 10-fold cross-validation [32], respectively equal to 0.66 and 8.5%.

A refined modeling approach, which allowed different taxa to respond differently by adding random intercepts and slopes for each predictor, one at a time, produced the best-fitting mixed-effect model. This model, with random effects for ^137^Cs and baseline abundance (Table 1), achieved higher GOF: conditional R^2^ = 0.657, suggesting that genus-specific responses to ^137^Cs helped to explain a lot of the variance. A visual comparison of model predictions with the data is shown in Fig 1 for two genera: *Arthrobacter*, which is common at Hanford and is radiosensitive, and *Deinococcus*, which was isolated only from one sample with high ^137^Cs levels and is radioresistant [13].

In the best-fitting mixed-effects model, the net regression coefficient for ^137^Cs (i.e. the sum of fixed and random effects) was negative for most bacterial genera (e.g. -0.0548 g/μCi for *Arthrobacter*), but positive (0.00878) for *Deinococcus*. *Agrococcus*, *Bacillus* and *Micrococcus* also had positive coefficients for ^137^Cs, although with smaller values than those for *Deinococcus*. These results imply that the most radioresistant taxa gained a competitive advantage over more radiosensitive ones in samples with high radioactivity levels.

### Data Set Two: Fungi in Chernobyl Reactor Buildings

This data set shows that multiple fungal taxa colonized the reactor buildings despite severe radioactive contamination and limited nutrient concentrations [18]. The bias-corrected Chao2 species richness estimator [26, 27] was essentially the same for low vs. high dose rate locations within the buildings: 44.3 (95% CI: 31.3, 108.7) and 44.8 (30.7, 113.4), respectively. Therefore, there was no evidence that the 15-fold increase in dose rate between the low and high dose rate locations [18] reduced species richness (for example by eliminating radiosensitive species and allowing radioresistant ones to fill vacated niches). This result suggests that even though the dose rate within the buildings was > 10^5^-fold higher than the natural background [18], it remained well below the critical value for population extinction for most/all of the 37 detected species.

Analysis of detection probabilities for each fungal species supported the same conclusion. These probabilities [18] were not significantly different (using Type I error threshold of 0.05) between low vs. high dose rate locations for any species, except for *Penicillium hirsutum*. This species was found in 8 out of 9 high dose rate samples and in only 1 out of 15 low dose rate samples. Therefore, the odds ratio for detection at high vs. low dose rates was 112 (95% CI: 4.61, 5290, P = 0.0001), suggesting that increased radiation (within the studied range of dose rates) made conditions more (rather than less) favorable for the growth of *P*. *hirsutum*.

Analysis of the data for all species using a GLMM suggested that most species isolated from the reactor buildings were more (rather than less) abundant in locations with high dose rates. This can be visualized by examining the observed and best-fit model-predicted odds ratios for detection of each species in high vs. low radiation locations (Fig 2). The predicted odds ratios were consistent with observed values/error bars and were > 1 for 32 out of 37 species (Fig 2).

The best-fit model coefficient for the fixed effect of increased radiation was positive, rather than negative, but not significantly different from zero: 0.35 (SE: 0.29, P = 0.23). This fixed effect explained very little of the variance: marginal R^2^ was 0.007. Random effects of radiation explained more, bringing conditional R^2^ to 0.285. However, this low value suggests that most of the variance remained unexplained and probably resulted from unmeasured factors: e.g. environmental variables other than radiation, interactions between fungal species, etc.

These results were not qualitatively altered when potential correlations between samples were investigated by merging data from randomly-selected samples into clusters of various sizes, and fitting the model to multiple synthetic data sets generated in this manner from the observed data set. The fixed effect of radiation fluctuated around zero, and the predicted odds ratios for detection in high vs. low radiation locations remained > 1 for several species.

At the most extreme case of inter-sample correlation (when the data from all samples at low dose rate were merged into a single presence/absence value and the same was done to all samples at high dose rate, reducing the data set to only two presence/absence values for each species), the coefficient for the fixed effect of increased radiation became negative, but not significantly different from zero: –0.66 (SE: 4.8, P = 0.89). Again, this fixed effect explained very little of the variance: marginal R^2^ was < 10^−4^. In contrast, random effects of order and species on the response to radiation explained most of the variance of this reduced data set, bringing conditional R^2^ to 0.998. Predicted odds ratios for detection in high vs. low radiation locations remained > 1 for 9 species: *Aspergillus flavus*, *A*. *fresenii*, *A*. *ochraceus*, *A*. *ustus*, *Beauveria bassiana*, *Geotrichum candidum*, *Paecilomyces variotii*, *Penicillium citrinum*, and *Phialophora melinii*. Consequently, the presence or absence of fungal taxa within Chernobyl reactor buildings was probably determined mainly by taxon-specific responses to radiation, which were positive rather than negative for several taxa.

The lethal dose of acute irradiation (in kGy), taken from reference [25], was associated with detection of a given taxon in the reactor buildings. When this variable was added to GLMM analysis, the coefficient relating it to fungal detection probability was 0.20 (SE: 0.058, P = 0.00038). However, resistance to acute irradiation did not predict differences in fungal abundance at high vs low dose rate locations: the coefficient for the interaction of radiation level with acute lethal dose was -0.04 (SE: 0.17, P = 0.81). This suggests that the most radioresistant species (*Alternaria alternata* and *Cladosporium cladosporioides*) were more likely to be found in the reactor buildings, than more sensitive species, but that their abundance was unaffected by dose rate fluctuations within the buildings.

### Data Set Three: Continuously Irradiated Yeast

**Fig 3:**
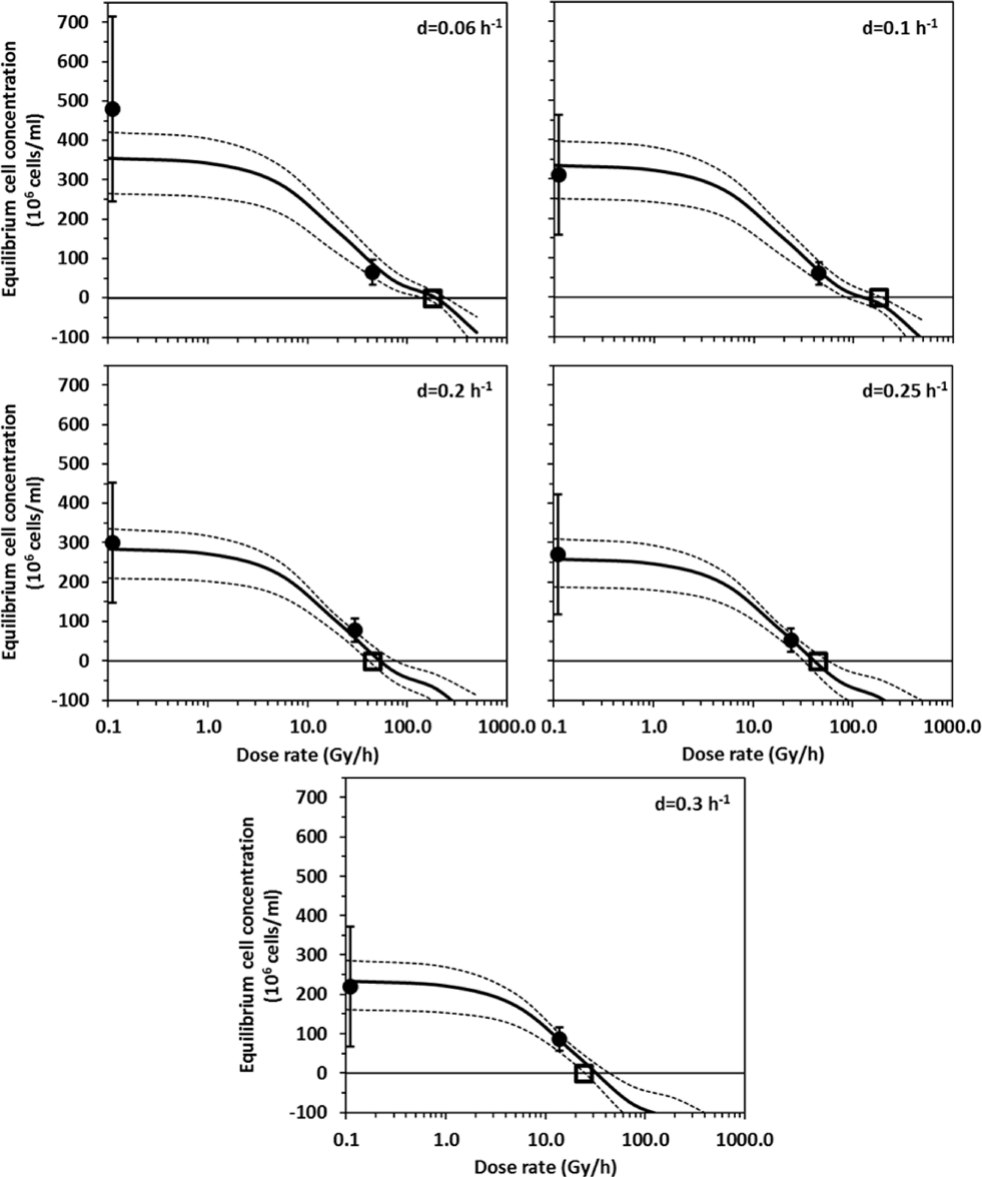
Comparison of best-fit model predictions (curves) with the data (symbols) on continuously irradiated yeast (data set three, reference [19], S1 Appendix) at various radiation dose rates and chemostat dilution rates (*d*). Solid circles are measurements of the equilibrium yeast cell concentration, which represents the steady-state number of cells/ml attained in the chemostat after multiple yeast generations (error bars are 95% CIs). Open squares are measurements of the critical dose rate above which the population became extinct. Solid curves are best-fit model predictions (from the best-supported model M_1_, Table 2). Dashed curves represent 95% CIs for model predictions, generated by fitting the model to multiple synthetic data sets generated from the original data set by Monte Carlo simulation (S4 Appendix). Predicted negative values for the equilibrium cell concentration (i.e. curves extending below zero on the y-axis) indicate population extinction. Points measured at 0 Gy/h are shown at 0.1 Gy/h so they can be displayed on a logarithmic scale.

This data set shows how the maximum radiation dose rate that a population can tolerate depends on another stressor–cell removal rate by chemostat dilution [19]. To analyze it, we used twenty mechanistically-motivated formalisms (summarized in Table 2). Nine of them (models M_1_–M_9_) had a fair degree of support from the data—based on values of the sample-size corrected Akaike information criterion (AICc) [33, 34]. The absence of overwhelming support for one model is not unexpected, given the overlap between mechanisms represented in different models (Table 2) and small data set size [19]. The best-supported formalism was M_1_ (Table 2), and it provided a reasonable fit to the data (Fig 3).

**Table 2:** Summary of mechanistic models used to analyze the data on continuously irradiated yeast (data set three, reference [19], S1 Appendix), arranged with the best-supported models at the top of the list. Details are provided in the main text and in S4 Appendix. Briefly, increasing ΔAICc and decreasing Akaike weight (*W*) indicate decreasing support. The description of models M_2_–M_20_ focuses on the features which are different for each model compared with model M_1_. The mathematical structure of each model is represented by (dN/dt)/N, which is the relative rate of change of the yeast cell concentration (N). R is the radiation dose rate and *d* is the chemostat dilution rate. Model parameters are *m*, *g*, *q*, *k*, δ, μ, σ. Not all parameters are present in every model, and the same symbol can represent parameters with different meaning in different models. *For a model which allowed different radiation effects on fast and slow cell proliferation components (using the structure (dN/dt)/N = –*d*–*m*×N+*g*×exp(–μ×R)+*q*×exp(–δ×R)–*k*×R) the best-fit value of parameter μ was 0, so the mathematical result was indistinguishable from model M_1_ and the Akaike weight was attributed to M_1_.

Model	Description	Mathematical expression for (dN/dt)/N	ΔAICc	*W*
M_1_	basic formalism with cell killing and suppression of proliferation by radiation	–*d*–*m*×N+*g*+*q*×exp(–δ×R)–*k*×R	0.00	0.544*
M_2_	radiation increases intraspecific competition	–*d*–*m*×N×exp(μ×R)+*g*+*q*×exp(–δ×R)–*k*×R	3.37	0.096
M_3_	dilution rate decreases radiation effect on cell proliferation	–*d*–*m*×N+*g*+*q*×exp(–δ×R)/(*d*×μ+1)–*k*×R	3.83	0.076
M_4_	radiation effect on cell proliferation is non-exponential	–*d*–*m*×N+*g*+*q*/(R×δ+1)–*k*×R	4.08	0.067
M_5_	dilution rate decreases cell killing by radiation	–*d*–*m*×N+*g*+*q*×exp(–δ×R)–*k*×R/(*d*×μ+1)	4.74	0.048
M_6_	cell killing by radiation is linear-quadratic	–*d*–*m*×N+*g*+*q*×exp(–δ×R)–μ×R^2^–*k*×R	5.29	0.037
M_7_	intraspecific competition is affected by interaction of dilution rate with radiation	–*d*–*m*×N×exp(–μ×*d*×R)+*g*+*q*×exp(–δ×R)–*k*×R	5.44	0.034
M_8_	radiation effect on cell proliferation is linear-quadratic	– *d*–*m*×N+*g*+*q*×exp(–R^2^×μ–R×δ)–*k*×R	5.52	0.033
M_9_	radiation stimulates cell proliferation at low dose rates	–*d*–*m*×N+*g*+(*q*+μ×R^1/2^)×exp(–δ×R)–*k*×R	5.81	0.028
M_10_	radiation increases intraspecific competition, but no radiation-independent cell proliferation rate component	–*d*–*m*×N×exp(μ×R)+q×exp(–δ×R)–k×R	7.02	0.015
M_11_	radiation increases intraspecific competition, but no cell killing by radiation	–*d*–*m*×N×exp(μ×R)+*g*+*q*×exp(–δ×R)	8.68	0.007
M_12_	radiation effect on intraspecific competition is linear-quadratic	–*d*–*m*×N×exp(R^2^×σ+R×μ)+*g*+*q*×exp(–δ×R)–*k*×R	8.72	0.007
M_13_	with separate non-exponential radiation effects on cell proliferation rate components	–*d*–*m*×N+*g*/(R×μ+1)+*q*/(R×δ+1)–*k*×R	9.91	0.004
M_14_	dilution rate decreases radiation effects on cell proliferation and intraspecific competition	–*d*–*m*×N/(*d*×μ+1)+*g*+*q*×exp(–δ×R)/(*d*×σ+1)–*k*×R	10.07	0.003
M_15_	with negative effect of dilution rate on cell killing by radiation and on intraspecific competition	–*d*–*m*×N/(*d*×μ+1)+*g*+*q*×exp(–δ×R)–*k*×R/(*d*×σ+1)	12.21	0.001
M_16_	radiation effects are linear-quadratic for cell killing and for proliferation	–*d*–*m*×N+*g*+*q*×exp(–R^2^×μ–R×δ)–σ×R^2^–*k*×R	12.75	0.001
M_17_	no direct killing by radiation	–*d*–*m*×N+*g*+*q*×exp(–δ×R)	18.61	0.000
M_18_	no radiation-independent cell proliferation rate component	–*d*–*m*×N+*q*×exp(–δ×R)–*k*×R	23.17	0.000
M_19_	radiation increases intraspecific competition, but no radiation effect on cell proliferation	–*d*–*m*×N×exp(μ×R)+*g*–*k*×R	26.86	0.000
M_20_	no radiation effect on cell proliferation	–*d*–*m*×N+*g*–*k*×R	33.28	0.000

Direct cell killing by radiation (parameter *k*) and suppression of cell proliferation by radiation (parameter δ) were very important for describing the data: if either of these parameters is excluded (models M_17_, M_19_, and M_20_, Table 2), support from the data will decrease virtually to zero. Interestingly, the radiation-independent cell proliferation rate component (parameter *g*) was also very important: if it is excluded (models M_10_ and M_18_, Table 2), model performance will become poor. This is consistent with published data, which show that the proliferation rate of continuously irradiated yeast stabilizes at a non-zero value even at very high dose rates [35]. Perhaps this occurs because of the interplay between accumulation and repair of radiation damage and checkpoint-induced proliferation arrest dynamics.

There was little support for quadratic terms for radiation effects on cell killing (models M_6_, M_16_, Table 2). This result is in agreement with radiobiological data and theory, which suggest that at low dose rates most DNA double strand breaks are rejoined before they can interact with each other, thereby creating a linear dose response [36, 37]. Quadratic terms or alternative dose response shapes for cell proliferation, including the possibility that low radiation dose rates stimulate cell proliferation (models M_4_, M_8_, M_9_, M_13_, M_16_, Table 2), also had low support. However, this finding may be in part explained by the small size of the data set, which causes complex models to be heavily penalized by AICc.

There was some support for effects of radiation and/or dilution rate on intraspecific competition: the sum of Akaike weights [33, 34] for models M_2_, M_7_, M_10_, M_11_, M_12_, M_14_ and M_15_ was 0.163. Furthermore, there was some evidence that dilution rate interacts with radiation: the sum of Akaike weights for models M_3_, M_5_, M_7_, M_14_, and M_15_ was 0.162. Perhaps these effects are caused by changes in the cell cycle phase/age structure of the irradiated yeast population [19, 38].

The parameter values for the best-supported model M_1_ were quite robust to random modification of the data set: *k* = cell killing by radiation, best-fit value 5.78 (95% CI: 3.14, 9.08)×10^−4^ Gy^-1^; δ = suppression of cell proliferation by radiation, 4.78 (3.27, 7.89)×10^−2^ h/Gy; *q* = fast (radiation-responsive) component of cell proliferation rate, 0.599 (0.410, 1.01) h^-1^; *m* = coefficient of intraspecific competition, 2.11 (1.43, 3.68)×10^−3^ ml/(h×10^6^ cells). The radiation-related parameters *k* and δ were highly correlated (*r* = 0.818), and so were non radiation-related parameters *q* and *m* (*r* = 0.832). As mentioned in S4 Appendix, parameter *g* was estimated analytically from data which were not fitted, so it was not freely adjustable.

**Fig 4:**
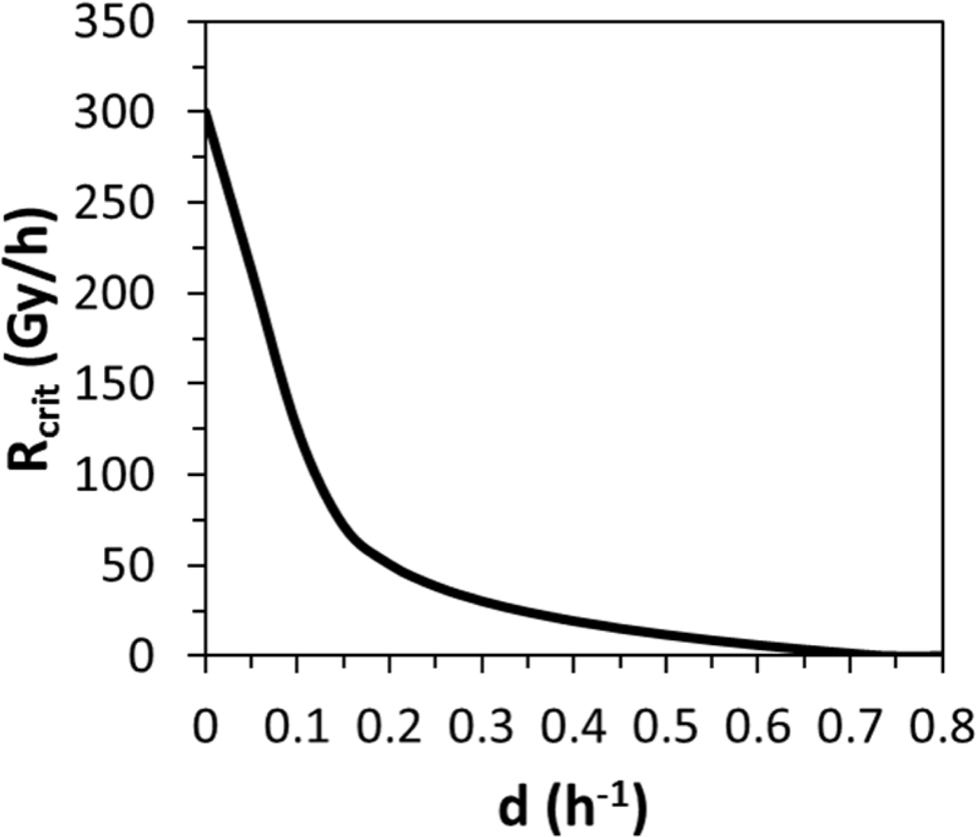
Best-fit prediction by MMI using all highly supported models (with ΔAICc < 6) for continuously irradiated yeast (data set three, reference [19], S1 Appendix). Details of the methods used are provided in S4 Appendix. The predicted variable is the critical radiation dose rate (R_crit_) above which the yeast population becomes extinct, as function of the chemostat dilution rate (*d*).

**Fig 5:**
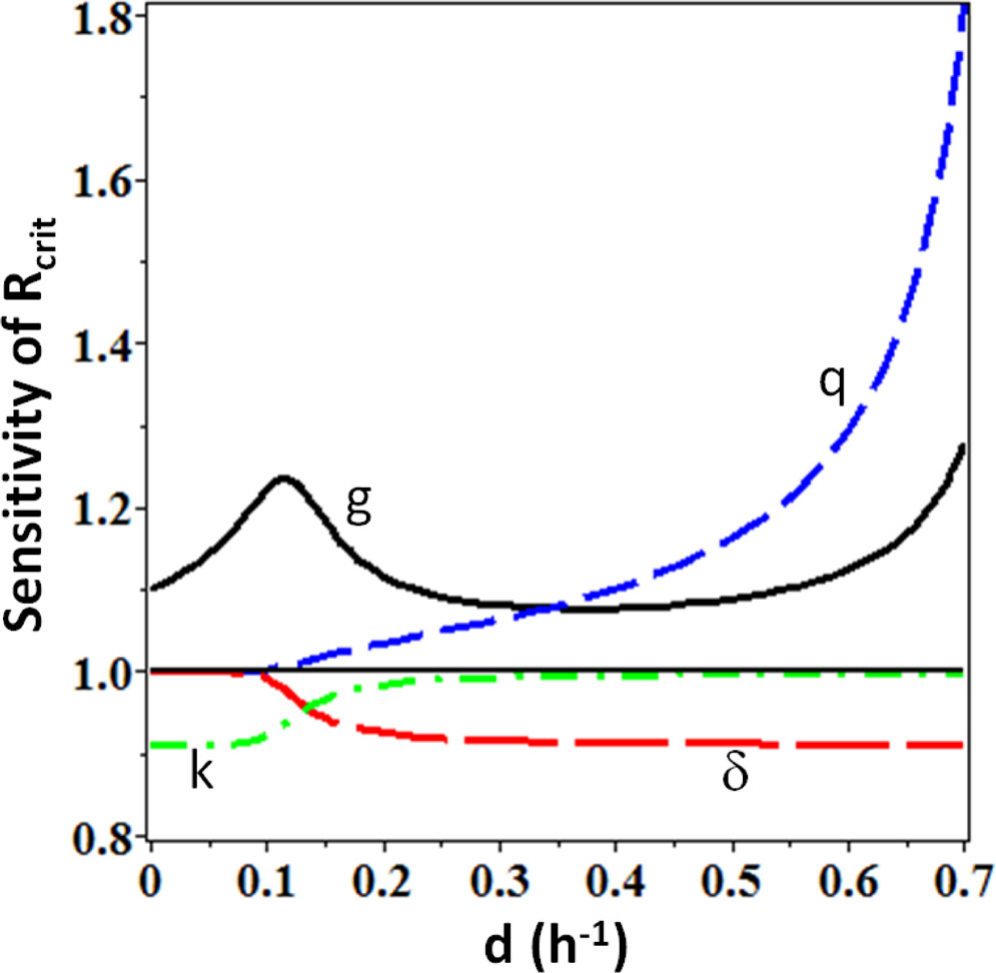
Sensitivity of the critical radiation dose rate (R_crit_) predicted by the best-supported model M_1_ (Table 2) for continuously irradiated yeast (data set three, reference [19], S1 Appendix) to a 10% increase in each of the model parameters (*g*, *q*, δ, *k*), as function of the chemostat dilution rate (*d*). When one parameter was increased, all others were held constant at best-fit values. The *y*-axis is the ratio of R_crit_ calculated when one of the parameters was increased to the value of R_crit_ calculated when all parameters were at their best-fit values. Details of the methods used are provided in S4 Appendix.

Combined predictions from all models generated by MMI were numerically very similar to those of the best-supported model M_1_. Consequently, both model M_1_ and MMI suggested the following conclusions:

1. The critical dose rate decreased rapidly and non-linearly with increasing dilution rate (Fig 4). For example, a dilution rate which caused 2-fold decrease in equilibrium cell concentration without irradiation decreased the critical dose rate by 13.3-fold. At sufficiently high dilution rates, extinction, of course, occurred even without excess irradiation.

1. The critical dose rate was sensitive to radiation-induced cell killing (parameter *k*) only at low dilution rates; whereas at high dilution rates effects on cell proliferation rate (parameters *q*, *g*, δ) became most influential (Fig 5). In other words, when the yeast population was subjected to combined stresses from radiation and dilution, the maximum tolerated dose rate was mainly determined by the ability to proliferate fast enough to compensate for cell loss by dilution. Thus, this experimental system serves as a simple example showing that radiation effects on suppression of reproduction can be more influential on populations subjected to multiple stressors than direct killing by radiation [39].

## Discussion

Taken together, the results of our analysis of three diverse data sets by descriptive and mechanistic mathematical modeling approaches demonstrate that microbial population responses to radioactive contamination can be strongly influenced by additional stressors. These stressors, such as chemical toxicants, should be taken into consideration when predicting radionuclide effects on the environment [6, 8, 9].

Specifically, analysis of data set one revealed that the nuclear waste at Hanford had strong toxic effects on soil bacteria. However, much of these effects were caused by chemical contaminants (e.g. NO_2_) rather than by radionuclides. Radiation from radionuclides (e.g. ^137^Cs) probably provided a competitive advantage for taxa with the highest resistance to radiation and oxidative stress (e.g. *Deinococcus*).

The results for data set two suggest that chronic irradiation can potentially produce non-monotonic dose response shapes: the probability to detect some (or even most) fungal species within the Chernobyl reactor buildings was predicted to increase (rather than decrease) with increasing radiation dose rate (within the range of dose rates represented in this data set). These findings are consistent with previously reported stimulation of directional growth and/or proliferation of some fungi by chronic irradiation [40–44].

Mutation rates in fungi within reactor buildings were probably elevated [45, 46], but it is unclear whether or not they exceeded the lethal mutagenesis threshold, which was measured only for some bacteria and viruses [47, 48]. Therefore, long-term genetic consequences of chronic irradiation for these fungal populations are unknown and could result either in progressive accumulation of deleterious mutations and extinction, or in accumulation of beneficial mutations [47, 48].

Analysis of data set three showed that the presence of additional stressors, such as a high cell mortality (removal) rate due to factors other than radiation, lowers by several-fold the critical radiation dose rate, which precipitates population extinction. In addition, reproduction suppression by radiation can be more important for determining the critical dose rate, than radiation-induced cell mortality.

The conclusions drawn from analyzing these three data sets quantitatively support the following generalizations, which could be useful for environmental protection and radionuclide bioremediation [6, 8].

First, the most severe effects (e.g. extinction) on microbial populations may occur when unfavorable environmental conditions (e.g. fluctuations of temperature and/or nutrient levels) coincide with radioactive contamination. For example, certain microbial species may persist in contaminated areas for some time, but become extinct when conditions become harsher due to seasonal and/or random factors. Consequently, to predict the long-term responses of such communities to radioactive contamination, it may be insufficient to measure radiation toxicity to the target organisms under laboratory conditions, or even under typical field conditions–measurements under the worst expected conditions (i.e. when non-radiation stressors attain maximal values) may be needed.

Second, to identify promising candidates for microbial bioremediation of radioactive wastes, it may be insufficient to screen only for radioresistance and/or efficiency in remediating certain chemicals–robustness against multiple stressors may be required to carry out bioremediation in the field. For example, many organisms which can grow in rich laboratory media with high radionuclide concentrations [9] may not be able to cope with similar concentrations in soil, where nutrient availability is lower and other stressors are present. Consequently, an organism’s ability to survive and proliferate under oligotrophic conditions in the presence of severe radioactive contamination would be needed to usefully exploit this organism for bioremediation [7]. Such abilities were demonstrated by several fungal taxa (e.g. *Penicillium*, *Cladosporium*, *Alternaria*) in data set two, suggesting that their potential as radionuclide bioremediators may need to be evaluated further [7].

The strengths of the present study include rigorous quantitative analysis intended to identify parsimonious explanations for how radiation and other stressors affected bacterial and fungal taxa under both field and laboratory conditions. State of the art descriptive and mechanistic methods, which incorporate elements of machine learning [49, 50] and information theory [33, 34], were employed.

The limitations of this study result from the properties of the data sets. For example, in data set one only a single contaminated site was sampled and, therefore, there is no guarantee that this site is representative. In data set two, many potentially relevant environmental variables (e.g. spatial locations of samples, temperature, pH) were not reported. In data set three, sample size was limited and results of replicate experiments at each stressor combination were not reported.

The limitations of the data sets resulted in unavoidable limitations of the methodology used to analyze them. In particular, the large number of taxa and/or stressors in data sets one and two prevented mechanistic modeling and we therefore relied on descriptive approaches, although in principle we believe that mechanistic models provide advantages over statistical ones [29, 30]. We had insufficient information about which taxa have important interactions with others, and therefore we had no choice but to ignore these interactions. Finally, the large number of potential predictor variables (radionuclides, chemical contaminants, temperature, pH, etc) in data set one prompted us to adopt a step-wise filter procedure to refine the data analysis. The goal of the filter procedure was to identify the most important predictors, but because not all possible predictor combinations were evaluated, there is no guarantee that the best-supported model was found.

Despite these limitations, we believe that our analysis of three diverse data sets provides useful insight into the important role of chemical and environmental stressors in determining the responses of microbial populations to radioactive contamination and might help to select the best microbial candidates for use in bioremediation.

## Supporting Information

S1 Appendix (DOCX)

S2 Appendix (DOCX)

S3 Appendix (DOCX)

S4 Appendix (DOCX)
