# Supplementary material for: Quantitative Modeling of Microbial Population Responses to Chronic Irradiation Combined with Other Stressors
Source: PLoS One. 2016 Jan 25;11(1):e0147696. doi: 10.1371/journal.pone.0147696 (PMC4726741; doi:10.1371/journal.pone.0147696)
Supplement: S1 Appendix — (DOCX) [file pone.0147696.s001.docx]

**S1 Appendix: Data Sets**

Data Set One: Bacteria at the Hanford Nuclear Waste Site

This data set was generated by bacteriological analysis of vadose sediments located under a high-level radioactive waste storage tank at the Hanford site, near Richland, Washington [1]. Temperature, pH, water content, conductivity, concentrations of ^137^Cs, ^99^Tc, Cr, NO_3_ and NO_2_, and the identities of detected bacterial taxa were recorded for each sample. We used the information presented mainly in Tables 1 and 3 and in Figures 1 and 2 of reference [1] to compile the following data set which consisted of binary (1 = presence, 0 = absence) values for each bacterial genus in each soil sample.

| **Genus** | **Pre-sence** | **Depth (m)** | **Water content (%)** | **pH** | **Conduc-tivity (mS/cm)** | **Tempe-rature (^o^C)** | **^137^Cs (1000× nGi/g)** | **^99^Tc (10^6^× pCi/L)** | **Cr (100x μg/g)** | **NO_3_ (1000x mg/L)** | **NO_2_ (mg/L)** |
| --- | --- | --- | --- | --- | --- | --- | --- | --- | --- | --- | --- |
| *Agrococcus* | 0 | 16.60 | 4.30 | 9.20 | 0.40 | 51.8 | 3.060 | 0.450 | 0.000 | 0.007 | 0.050 |
| *Alcaligenes* | 0 | 16.60 | 4.30 | 9.20 | 0.40 | 51.8 | 3.060 | 0.450 | 0.000 | 0.007 | 0.050 |
| *Arthrobacter* | 1 | 16.60 | 4.30 | 9.20 | 0.40 | 51.8 | 3.060 | 0.450 | 0.000 | 0.007 | 0.050 |
| *Azospirillum* | 0 | 16.60 | 4.30 | 9.20 | 0.40 | 51.8 | 3.060 | 0.450 | 0.000 | 0.007 | 0.050 |
| *Bacillus* | 0 | 16.60 | 4.30 | 9.20 | 0.40 | 51.8 | 3.060 | 0.450 | 0.000 | 0.007 | 0.050 |
| *Bradyrhizobium* | 0 | 16.60 | 4.30 | 9.20 | 0.40 | 51.8 | 3.060 | 0.450 | 0.000 | 0.007 | 0.050 |
| *Clavibacter* | 0 | 16.60 | 4.30 | 9.20 | 0.40 | 51.8 | 3.060 | 0.450 | 0.000 | 0.007 | 0.050 |
| *Deinococcus* | 0 | 16.60 | 4.30 | 9.20 | 0.40 | 51.8 | 3.060 | 0.450 | 0.000 | 0.007 | 0.050 |
| *Dermabacter* | 0 | 16.60 | 4.30 | 9.20 | 0.40 | 51.8 | 3.060 | 0.450 | 0.000 | 0.007 | 0.050 |
| *Detolaasinbacter* | 0 | 16.60 | 4.30 | 9.20 | 0.40 | 51.8 | 3.060 | 0.450 | 0.000 | 0.007 | 0.050 |
| *Janibacter* | 0 | 16.60 | 4.30 | 9.20 | 0.40 | 51.8 | 3.060 | 0.450 | 0.000 | 0.007 | 0.050 |
| *Microbacterium* | 0 | 16.60 | 4.30 | 9.20 | 0.40 | 51.8 | 3.060 | 0.450 | 0.000 | 0.007 | 0.050 |
| *Micrococcus* | 0 | 16.60 | 4.30 | 9.20 | 0.40 | 51.8 | 3.060 | 0.450 | 0.000 | 0.007 | 0.050 |
| *Mycobacterium* | 0 | 16.60 | 4.30 | 9.20 | 0.40 | 51.8 | 3.060 | 0.450 | 0.000 | 0.007 | 0.050 |
| *Nocardia* | 0 | 16.60 | 4.30 | 9.20 | 0.40 | 51.8 | 3.060 | 0.450 | 0.000 | 0.007 | 0.050 |
| *Nocardioides* | 0 | 16.60 | 4.30 | 9.20 | 0.40 | 51.8 | 3.060 | 0.450 | 0.000 | 0.007 | 0.050 |
| *proteobacteria* | 0 | 16.60 | 4.30 | 9.20 | 0.40 | 51.8 | 3.060 | 0.450 | 0.000 | 0.007 | 0.050 |
| *Pseudomonas* | 0 | 16.60 | 4.30 | 9.20 | 0.40 | 51.8 | 3.060 | 0.450 | 0.000 | 0.007 | 0.050 |
| *Rhodococcus* | 0 | 16.60 | 4.30 | 9.20 | 0.40 | 51.8 | 3.060 | 0.450 | 0.000 | 0.007 | 0.050 |
| *Sphingomonas* | 0 | 16.60 | 4.30 | 9.20 | 0.40 | 51.8 | 3.060 | 0.450 | 0.000 | 0.007 | 0.050 |
| *Staphylococcus* | 0 | 16.60 | 4.30 | 9.20 | 0.40 | 51.8 | 3.060 | 0.450 | 0.000 | 0.007 | 0.050 |
| *Stenotrophomonas* | 0 | 16.60 | 4.30 | 9.20 | 0.40 | 51.8 | 3.060 | 0.450 | 0.000 | 0.007 | 0.050 |
| *Streptomyces* | 0 | 16.60 | 4.30 | 9.20 | 0.40 | 51.8 | 3.060 | 0.450 | 0.000 | 0.007 | 0.050 |
| *Terrabacter* | 0 | 16.60 | 4.30 | 9.20 | 0.40 | 51.8 | 3.060 | 0.450 | 0.000 | 0.007 | 0.050 |
| *Variovorax* | 0 | 16.60 | 4.30 | 9.20 | 0.40 | 51.8 | 3.060 | 0.450 | 0.000 | 0.007 | 0.050 |
| *Verrucosispora* | 0 | 16.60 | 4.30 | 9.20 | 0.40 | 51.8 | 3.060 | 0.450 | 0.000 | 0.007 | 0.050 |
| *Agrococcus* | 0 | 20.50 | 2.80 | 9.60 | 0.70 | 60.8 | 19.500 | 0.134 | 0.010 | 0.029 | 0.400 |
| *Alcaligenes* | 0 | 20.50 | 2.80 | 9.60 | 0.70 | 60.8 | 19.500 | 0.134 | 0.010 | 0.029 | 0.400 |
| *Arthrobacter* | 0 | 20.50 | 2.80 | 9.60 | 0.70 | 60.8 | 19.500 | 0.134 | 0.010 | 0.029 | 0.400 |
| *Azospirillum* | 0 | 20.50 | 2.80 | 9.60 | 0.70 | 60.8 | 19.500 | 0.134 | 0.010 | 0.029 | 0.400 |
| *Bacillus* | 0 | 20.50 | 2.80 | 9.60 | 0.70 | 60.8 | 19.500 | 0.134 | 0.010 | 0.029 | 0.400 |
| *Bradyrhizobium* | 0 | 20.50 | 2.80 | 9.60 | 0.70 | 60.8 | 19.500 | 0.134 | 0.010 | 0.029 | 0.400 |
| *Clavibacter* | 0 | 20.50 | 2.80 | 9.60 | 0.70 | 60.8 | 19.500 | 0.134 | 0.010 | 0.029 | 0.400 |
| *Deinococcus* | 0 | 20.50 | 2.80 | 9.60 | 0.70 | 60.8 | 19.500 | 0.134 | 0.010 | 0.029 | 0.400 |
| *Dermabacter* | 0 | 20.50 | 2.80 | 9.60 | 0.70 | 60.8 | 19.500 | 0.134 | 0.010 | 0.029 | 0.400 |
| *Detolaasinbacter* | 0 | 20.50 | 2.80 | 9.60 | 0.70 | 60.8 | 19.500 | 0.134 | 0.010 | 0.029 | 0.400 |
| *Janibacter* | 0 | 20.50 | 2.80 | 9.60 | 0.70 | 60.8 | 19.500 | 0.134 | 0.010 | 0.029 | 0.400 |
| *Microbacterium* | 0 | 20.50 | 2.80 | 9.60 | 0.70 | 60.8 | 19.500 | 0.134 | 0.010 | 0.029 | 0.400 |
| *Micrococcus* | 0 | 20.50 | 2.80 | 9.60 | 0.70 | 60.8 | 19.500 | 0.134 | 0.010 | 0.029 | 0.400 |
| *Mycobacterium* | 0 | 20.50 | 2.80 | 9.60 | 0.70 | 60.8 | 19.500 | 0.134 | 0.010 | 0.029 | 0.400 |
| *Nocardia* | 0 | 20.50 | 2.80 | 9.60 | 0.70 | 60.8 | 19.500 | 0.134 | 0.010 | 0.029 | 0.400 |
| *Nocardioides* | 0 | 20.50 | 2.80 | 9.60 | 0.70 | 60.8 | 19.500 | 0.134 | 0.010 | 0.029 | 0.400 |
| *proteobacteria* | 0 | 20.50 | 2.80 | 9.60 | 0.70 | 60.8 | 19.500 | 0.134 | 0.010 | 0.029 | 0.400 |
| *Pseudomonas* | 0 | 20.50 | 2.80 | 9.60 | 0.70 | 60.8 | 19.500 | 0.134 | 0.010 | 0.029 | 0.400 |
| *Rhodococcus* | 0 | 20.50 | 2.80 | 9.60 | 0.70 | 60.8 | 19.500 | 0.134 | 0.010 | 0.029 | 0.400 |
| *Sphingomonas* | 0 | 20.50 | 2.80 | 9.60 | 0.70 | 60.8 | 19.500 | 0.134 | 0.010 | 0.029 | 0.400 |
| *Staphylococcus* | 0 | 20.50 | 2.80 | 9.60 | 0.70 | 60.8 | 19.500 | 0.134 | 0.010 | 0.029 | 0.400 |
| *Stenotrophomonas* | 0 | 20.50 | 2.80 | 9.60 | 0.70 | 60.8 | 19.500 | 0.134 | 0.010 | 0.029 | 0.400 |
| *Streptomyces* | 0 | 20.50 | 2.80 | 9.60 | 0.70 | 60.8 | 19.500 | 0.134 | 0.010 | 0.029 | 0.400 |
| *Terrabacter* | 0 | 20.50 | 2.80 | 9.60 | 0.70 | 60.8 | 19.500 | 0.134 | 0.010 | 0.029 | 0.400 |
| *Variovorax* | 0 | 20.50 | 2.80 | 9.60 | 0.70 | 60.8 | 19.500 | 0.134 | 0.010 | 0.029 | 0.400 |
| *Verrucosispora* | 0 | 20.50 | 2.80 | 9.60 | 0.70 | 60.8 | 19.500 | 0.134 | 0.010 | 0.029 | 0.400 |
| *Agrococcus* | 0 | 21.80 | 2.80 | 9.50 | 0.58 | 63.8 | 1.380 | 0.057 | 0.009 | 0.024 | 0.300 |
| *Alcaligenes* | 0 | 21.80 | 2.80 | 9.50 | 0.58 | 63.8 | 1.380 | 0.057 | 0.009 | 0.024 | 0.300 |
| *Arthrobacter* | 0 | 21.80 | 2.80 | 9.50 | 0.58 | 63.8 | 1.380 | 0.057 | 0.009 | 0.024 | 0.300 |
| *Azospirillum* | 0 | 21.80 | 2.80 | 9.50 | 0.58 | 63.8 | 1.380 | 0.057 | 0.009 | 0.024 | 0.300 |
| *Bacillus* | 0 | 21.80 | 2.80 | 9.50 | 0.58 | 63.8 | 1.380 | 0.057 | 0.009 | 0.024 | 0.300 |
| *Bradyrhizobium* | 0 | 21.80 | 2.80 | 9.50 | 0.58 | 63.8 | 1.380 | 0.057 | 0.009 | 0.024 | 0.300 |
| *Clavibacter* | 1 | 21.80 | 2.80 | 9.50 | 0.58 | 63.8 | 1.380 | 0.057 | 0.009 | 0.024 | 0.300 |
| *Deinococcus* | 0 | 21.80 | 2.80 | 9.50 | 0.58 | 63.8 | 1.380 | 0.057 | 0.009 | 0.024 | 0.300 |
| *Dermabacter* | 0 | 21.80 | 2.80 | 9.50 | 0.58 | 63.8 | 1.380 | 0.057 | 0.009 | 0.024 | 0.300 |
| *Detolaasinbacter* | 0 | 21.80 | 2.80 | 9.50 | 0.58 | 63.8 | 1.380 | 0.057 | 0.009 | 0.024 | 0.300 |
| *Janibacter* | 0 | 21.80 | 2.80 | 9.50 | 0.58 | 63.8 | 1.380 | 0.057 | 0.009 | 0.024 | 0.300 |
| *Microbacterium* | 1 | 21.80 | 2.80 | 9.50 | 0.58 | 63.8 | 1.380 | 0.057 | 0.009 | 0.024 | 0.300 |
| *Micrococcus* | 0 | 21.80 | 2.80 | 9.50 | 0.58 | 63.8 | 1.380 | 0.057 | 0.009 | 0.024 | 0.300 |
| *Mycobacterium* | 0 | 21.80 | 2.80 | 9.50 | 0.58 | 63.8 | 1.380 | 0.057 | 0.009 | 0.024 | 0.300 |
| *Nocardia* | 1 | 21.80 | 2.80 | 9.50 | 0.58 | 63.8 | 1.380 | 0.057 | 0.009 | 0.024 | 0.300 |
| *Nocardioides* | 1 | 21.80 | 2.80 | 9.50 | 0.58 | 63.8 | 1.380 | 0.057 | 0.009 | 0.024 | 0.300 |
| *proteobacteria* | 0 | 21.80 | 2.80 | 9.50 | 0.58 | 63.8 | 1.380 | 0.057 | 0.009 | 0.024 | 0.300 |
| *Pseudomonas* | 0 | 21.80 | 2.80 | 9.50 | 0.58 | 63.8 | 1.380 | 0.057 | 0.009 | 0.024 | 0.300 |
| *Rhodococcus* | 1 | 21.80 | 2.80 | 9.50 | 0.58 | 63.8 | 1.380 | 0.057 | 0.009 | 0.024 | 0.300 |
| *Sphingomonas* | 0 | 21.80 | 2.80 | 9.50 | 0.58 | 63.8 | 1.380 | 0.057 | 0.009 | 0.024 | 0.300 |
| *Staphylococcus* | 1 | 21.80 | 2.80 | 9.50 | 0.58 | 63.8 | 1.380 | 0.057 | 0.009 | 0.024 | 0.300 |
| *Stenotrophomonas* | 0 | 21.80 | 2.80 | 9.50 | 0.58 | 63.8 | 1.380 | 0.057 | 0.009 | 0.024 | 0.300 |
| *Streptomyces* | 0 | 21.80 | 2.80 | 9.50 | 0.58 | 63.8 | 1.380 | 0.057 | 0.009 | 0.024 | 0.300 |
| *Terrabacter* | 0 | 21.80 | 2.80 | 9.50 | 0.58 | 63.8 | 1.380 | 0.057 | 0.009 | 0.024 | 0.300 |
| *Variovorax* | 0 | 21.80 | 2.80 | 9.50 | 0.58 | 63.8 | 1.380 | 0.057 | 0.009 | 0.024 | 0.300 |
| *Verrucosispora* | 0 | 21.80 | 2.80 | 9.50 | 0.58 | 63.8 | 1.380 | 0.057 | 0.009 | 0.024 | 0.300 |
| *Agrococcus* | 1 | 23.10 | 4.70 | 9.80 | 0.88 | 65.4 | 6.520 | 0.225 | 0.036 | 0.093 | 0.300 |
| *Alcaligenes* | 0 | 23.10 | 4.70 | 9.80 | 0.88 | 65.4 | 6.520 | 0.225 | 0.036 | 0.093 | 0.300 |
| *Arthrobacter* | 1 | 23.10 | 4.70 | 9.80 | 0.88 | 65.4 | 6.520 | 0.225 | 0.036 | 0.093 | 0.300 |
| *Azospirillum* | 0 | 23.10 | 4.70 | 9.80 | 0.88 | 65.4 | 6.520 | 0.225 | 0.036 | 0.093 | 0.300 |
| *Bacillus* | 1 | 23.10 | 4.70 | 9.80 | 0.88 | 65.4 | 6.520 | 0.225 | 0.036 | 0.093 | 0.300 |
| *Bradyrhizobium* | 0 | 23.10 | 4.70 | 9.80 | 0.88 | 65.4 | 6.520 | 0.225 | 0.036 | 0.093 | 0.300 |
| *Clavibacter* | 0 | 23.10 | 4.70 | 9.80 | 0.88 | 65.4 | 6.520 | 0.225 | 0.036 | 0.093 | 0.300 |
| *Deinococcus* | 0 | 23.10 | 4.70 | 9.80 | 0.88 | 65.4 | 6.520 | 0.225 | 0.036 | 0.093 | 0.300 |
| *Dermabacter* | 0 | 23.10 | 4.70 | 9.80 | 0.88 | 65.4 | 6.520 | 0.225 | 0.036 | 0.093 | 0.300 |
| *Detolaasinbacter* | 0 | 23.10 | 4.70 | 9.80 | 0.88 | 65.4 | 6.520 | 0.225 | 0.036 | 0.093 | 0.300 |
| *Janibacter* | 0 | 23.10 | 4.70 | 9.80 | 0.88 | 65.4 | 6.520 | 0.225 | 0.036 | 0.093 | 0.300 |
| *Microbacterium* | 0 | 23.10 | 4.70 | 9.80 | 0.88 | 65.4 | 6.520 | 0.225 | 0.036 | 0.093 | 0.300 |
| *Micrococcus* | 0 | 23.10 | 4.70 | 9.80 | 0.88 | 65.4 | 6.520 | 0.225 | 0.036 | 0.093 | 0.300 |
| *Mycobacterium* | 0 | 23.10 | 4.70 | 9.80 | 0.88 | 65.4 | 6.520 | 0.225 | 0.036 | 0.093 | 0.300 |
| *Nocardia* | 0 | 23.10 | 4.70 | 9.80 | 0.88 | 65.4 | 6.520 | 0.225 | 0.036 | 0.093 | 0.300 |
| *Nocardioides* | 0 | 23.10 | 4.70 | 9.80 | 0.88 | 65.4 | 6.520 | 0.225 | 0.036 | 0.093 | 0.300 |
| *proteobacteria* | 0 | 23.10 | 4.70 | 9.80 | 0.88 | 65.4 | 6.520 | 0.225 | 0.036 | 0.093 | 0.300 |
| *Pseudomonas* | 0 | 23.10 | 4.70 | 9.80 | 0.88 | 65.4 | 6.520 | 0.225 | 0.036 | 0.093 | 0.300 |
| *Rhodococcus* | 0 | 23.10 | 4.70 | 9.80 | 0.88 | 65.4 | 6.520 | 0.225 | 0.036 | 0.093 | 0.300 |
| *Sphingomonas* | 0 | 23.10 | 4.70 | 9.80 | 0.88 | 65.4 | 6.520 | 0.225 | 0.036 | 0.093 | 0.300 |
| *Staphylococcus* | 0 | 23.10 | 4.70 | 9.80 | 0.88 | 65.4 | 6.520 | 0.225 | 0.036 | 0.093 | 0.300 |
| *Stenotrophomonas* | 0 | 23.10 | 4.70 | 9.80 | 0.88 | 65.4 | 6.520 | 0.225 | 0.036 | 0.093 | 0.300 |
| *Streptomyces* | 0 | 23.10 | 4.70 | 9.80 | 0.88 | 65.4 | 6.520 | 0.225 | 0.036 | 0.093 | 0.300 |
| *Terrabacter* | 0 | 23.10 | 4.70 | 9.80 | 0.88 | 65.4 | 6.520 | 0.225 | 0.036 | 0.093 | 0.300 |
| *Variovorax* | 0 | 23.10 | 4.70 | 9.80 | 0.88 | 65.4 | 6.520 | 0.225 | 0.036 | 0.093 | 0.300 |
| *Verrucosispora* | 0 | 23.10 | 4.70 | 9.80 | 0.88 | 65.4 | 6.520 | 0.225 | 0.036 | 0.093 | 0.300 |
| *Agrococcus* | 0 | 24.40 | 3.70 | 8.00 | 16.71 | 65.5 | 53.100 | 1.184 | 4.838 | 11.740 | 0.050 |
| *Alcaligenes* | 0 | 24.40 | 3.70 | 8.00 | 16.71 | 65.5 | 53.100 | 1.184 | 4.838 | 11.740 | 0.050 |
| *Arthrobacter* | 0 | 24.40 | 3.70 | 8.00 | 16.71 | 65.5 | 53.100 | 1.184 | 4.838 | 11.740 | 0.050 |
| *Azospirillum* | 0 | 24.40 | 3.70 | 8.00 | 16.71 | 65.5 | 53.100 | 1.184 | 4.838 | 11.740 | 0.050 |
| *Bacillus* | 0 | 24.40 | 3.70 | 8.00 | 16.71 | 65.5 | 53.100 | 1.184 | 4.838 | 11.740 | 0.050 |
| *Bradyrhizobium* | 0 | 24.40 | 3.70 | 8.00 | 16.71 | 65.5 | 53.100 | 1.184 | 4.838 | 11.740 | 0.050 |
| *Clavibacter* | 0 | 24.40 | 3.70 | 8.00 | 16.71 | 65.5 | 53.100 | 1.184 | 4.838 | 11.740 | 0.050 |
| *Deinococcus* | 0 | 24.40 | 3.70 | 8.00 | 16.71 | 65.5 | 53.100 | 1.184 | 4.838 | 11.740 | 0.050 |
| *Dermabacter* | 0 | 24.40 | 3.70 | 8.00 | 16.71 | 65.5 | 53.100 | 1.184 | 4.838 | 11.740 | 0.050 |
| *Detolaasinbacter* | 0 | 24.40 | 3.70 | 8.00 | 16.71 | 65.5 | 53.100 | 1.184 | 4.838 | 11.740 | 0.050 |
| *Janibacter* | 0 | 24.40 | 3.70 | 8.00 | 16.71 | 65.5 | 53.100 | 1.184 | 4.838 | 11.740 | 0.050 |
| *Microbacterium* | 0 | 24.40 | 3.70 | 8.00 | 16.71 | 65.5 | 53.100 | 1.184 | 4.838 | 11.740 | 0.050 |
| *Micrococcus* | 0 | 24.40 | 3.70 | 8.00 | 16.71 | 65.5 | 53.100 | 1.184 | 4.838 | 11.740 | 0.050 |
| *Mycobacterium* | 0 | 24.40 | 3.70 | 8.00 | 16.71 | 65.5 | 53.100 | 1.184 | 4.838 | 11.740 | 0.050 |
| *Nocardia* | 0 | 24.40 | 3.70 | 8.00 | 16.71 | 65.5 | 53.100 | 1.184 | 4.838 | 11.740 | 0.050 |
| *Nocardioides* | 0 | 24.40 | 3.70 | 8.00 | 16.71 | 65.5 | 53.100 | 1.184 | 4.838 | 11.740 | 0.050 |
| *proteobacteria* | 0 | 24.40 | 3.70 | 8.00 | 16.71 | 65.5 | 53.100 | 1.184 | 4.838 | 11.740 | 0.050 |
| *Pseudomonas* | 0 | 24.40 | 3.70 | 8.00 | 16.71 | 65.5 | 53.100 | 1.184 | 4.838 | 11.740 | 0.050 |
| *Rhodococcus* | 0 | 24.40 | 3.70 | 8.00 | 16.71 | 65.5 | 53.100 | 1.184 | 4.838 | 11.740 | 0.050 |
| *Sphingomonas* | 0 | 24.40 | 3.70 | 8.00 | 16.71 | 65.5 | 53.100 | 1.184 | 4.838 | 11.740 | 0.050 |
| *Staphylococcus* | 0 | 24.40 | 3.70 | 8.00 | 16.71 | 65.5 | 53.100 | 1.184 | 4.838 | 11.740 | 0.050 |
| *Stenotrophomonas* | 0 | 24.40 | 3.70 | 8.00 | 16.71 | 65.5 | 53.100 | 1.184 | 4.838 | 11.740 | 0.050 |
| *Streptomyces* | 0 | 24.40 | 3.70 | 8.00 | 16.71 | 65.5 | 53.100 | 1.184 | 4.838 | 11.740 | 0.050 |
| *Terrabacter* | 0 | 24.40 | 3.70 | 8.00 | 16.71 | 65.5 | 53.100 | 1.184 | 4.838 | 11.740 | 0.050 |
| *Variovorax* | 0 | 24.40 | 3.70 | 8.00 | 16.71 | 65.5 | 53.100 | 1.184 | 4.838 | 11.740 | 0.050 |
| *Verrucosispora* | 0 | 24.40 | 3.70 | 8.00 | 16.71 | 65.5 | 53.100 | 1.184 | 4.838 | 11.740 | 0.050 |
| *Agrococcus* | 0 | 25.60 | 6.20 | 9.60 | 54.62 | 73.1 | 21.400 | 15.271 | 3.097 | 46.640 | 0.050 |
| *Alcaligenes* | 0 | 25.60 | 6.20 | 9.60 | 54.62 | 73.1 | 21.400 | 15.271 | 3.097 | 46.640 | 0.050 |
| *Arthrobacter* | 0 | 25.60 | 6.20 | 9.60 | 54.62 | 73.1 | 21.400 | 15.271 | 3.097 | 46.640 | 0.050 |
| *Azospirillum* | 0 | 25.60 | 6.20 | 9.60 | 54.62 | 73.1 | 21.400 | 15.271 | 3.097 | 46.640 | 0.050 |
| *Bacillus* | 0 | 25.60 | 6.20 | 9.60 | 54.62 | 73.1 | 21.400 | 15.271 | 3.097 | 46.640 | 0.050 |
| *Bradyrhizobium* | 0 | 25.60 | 6.20 | 9.60 | 54.62 | 73.1 | 21.400 | 15.271 | 3.097 | 46.640 | 0.050 |
| *Clavibacter* | 0 | 25.60 | 6.20 | 9.60 | 54.62 | 73.1 | 21.400 | 15.271 | 3.097 | 46.640 | 0.050 |
| *Deinococcus* | 1 | 25.60 | 6.20 | 9.60 | 54.62 | 73.1 | 21.400 | 15.271 | 3.097 | 46.640 | 0.050 |
| *Dermabacter* | 0 | 25.60 | 6.20 | 9.60 | 54.62 | 73.1 | 21.400 | 15.271 | 3.097 | 46.640 | 0.050 |
| *Detolaasinbacter* | 0 | 25.60 | 6.20 | 9.60 | 54.62 | 73.1 | 21.400 | 15.271 | 3.097 | 46.640 | 0.050 |
| *Janibacter* | 0 | 25.60 | 6.20 | 9.60 | 54.62 | 73.1 | 21.400 | 15.271 | 3.097 | 46.640 | 0.050 |
| *Microbacterium* | 0 | 25.60 | 6.20 | 9.60 | 54.62 | 73.1 | 21.400 | 15.271 | 3.097 | 46.640 | 0.050 |
| *Micrococcus* | 1 | 25.60 | 6.20 | 9.60 | 54.62 | 73.1 | 21.400 | 15.271 | 3.097 | 46.640 | 0.050 |
| *Mycobacterium* | 0 | 25.60 | 6.20 | 9.60 | 54.62 | 73.1 | 21.400 | 15.271 | 3.097 | 46.640 | 0.050 |
| *Nocardia* | 0 | 25.60 | 6.20 | 9.60 | 54.62 | 73.1 | 21.400 | 15.271 | 3.097 | 46.640 | 0.050 |
| *Nocardioides* | 0 | 25.60 | 6.20 | 9.60 | 54.62 | 73.1 | 21.400 | 15.271 | 3.097 | 46.640 | 0.050 |
| *proteobacteria* | 0 | 25.60 | 6.20 | 9.60 | 54.62 | 73.1 | 21.400 | 15.271 | 3.097 | 46.640 | 0.050 |
| *Pseudomonas* | 0 | 25.60 | 6.20 | 9.60 | 54.62 | 73.1 | 21.400 | 15.271 | 3.097 | 46.640 | 0.050 |
| *Rhodococcus* | 0 | 25.60 | 6.20 | 9.60 | 54.62 | 73.1 | 21.400 | 15.271 | 3.097 | 46.640 | 0.050 |
| *Sphingomonas* | 0 | 25.60 | 6.20 | 9.60 | 54.62 | 73.1 | 21.400 | 15.271 | 3.097 | 46.640 | 0.050 |
| *Staphylococcus* | 0 | 25.60 | 6.20 | 9.60 | 54.62 | 73.1 | 21.400 | 15.271 | 3.097 | 46.640 | 0.050 |
| *Stenotrophomonas* | 0 | 25.60 | 6.20 | 9.60 | 54.62 | 73.1 | 21.400 | 15.271 | 3.097 | 46.640 | 0.050 |
| *Streptomyces* | 0 | 25.60 | 6.20 | 9.60 | 54.62 | 73.1 | 21.400 | 15.271 | 3.097 | 46.640 | 0.050 |
| *Terrabacter* | 0 | 25.60 | 6.20 | 9.60 | 54.62 | 73.1 | 21.400 | 15.271 | 3.097 | 46.640 | 0.050 |
| *Variovorax* | 0 | 25.60 | 6.20 | 9.60 | 54.62 | 73.1 | 21.400 | 15.271 | 3.097 | 46.640 | 0.050 |
| *Verrucosispora* | 0 | 25.60 | 6.20 | 9.60 | 54.62 | 73.1 | 21.400 | 15.271 | 3.097 | 46.640 | 0.050 |
| *Agrococcus* | 0 | 26.90 | 6.00 | 7.90 | 49.01 | 74.3 | 0.555 | 47.772 | 8.298 | 39.710 | 87.500 |
| *Alcaligenes* | 0 | 26.90 | 6.00 | 7.90 | 49.01 | 74.3 | 0.555 | 47.772 | 8.298 | 39.710 | 87.500 |
| *Arthrobacter* | 0 | 26.90 | 6.00 | 7.90 | 49.01 | 74.3 | 0.555 | 47.772 | 8.298 | 39.710 | 87.500 |
| *Azospirillum* | 0 | 26.90 | 6.00 | 7.90 | 49.01 | 74.3 | 0.555 | 47.772 | 8.298 | 39.710 | 87.500 |
| *Bacillus* | 0 | 26.90 | 6.00 | 7.90 | 49.01 | 74.3 | 0.555 | 47.772 | 8.298 | 39.710 | 87.500 |
| *Bradyrhizobium* | 0 | 26.90 | 6.00 | 7.90 | 49.01 | 74.3 | 0.555 | 47.772 | 8.298 | 39.710 | 87.500 |
| *Clavibacter* | 0 | 26.90 | 6.00 | 7.90 | 49.01 | 74.3 | 0.555 | 47.772 | 8.298 | 39.710 | 87.500 |
| *Deinococcus* | 0 | 26.90 | 6.00 | 7.90 | 49.01 | 74.3 | 0.555 | 47.772 | 8.298 | 39.710 | 87.500 |
| *Dermabacter* | 0 | 26.90 | 6.00 | 7.90 | 49.01 | 74.3 | 0.555 | 47.772 | 8.298 | 39.710 | 87.500 |
| *Detolaasinbacter* | 0 | 26.90 | 6.00 | 7.90 | 49.01 | 74.3 | 0.555 | 47.772 | 8.298 | 39.710 | 87.500 |
| *Janibacter* | 0 | 26.90 | 6.00 | 7.90 | 49.01 | 74.3 | 0.555 | 47.772 | 8.298 | 39.710 | 87.500 |
| *Microbacterium* | 0 | 26.90 | 6.00 | 7.90 | 49.01 | 74.3 | 0.555 | 47.772 | 8.298 | 39.710 | 87.500 |
| *Micrococcus* | 0 | 26.90 | 6.00 | 7.90 | 49.01 | 74.3 | 0.555 | 47.772 | 8.298 | 39.710 | 87.500 |
| *Mycobacterium* | 0 | 26.90 | 6.00 | 7.90 | 49.01 | 74.3 | 0.555 | 47.772 | 8.298 | 39.710 | 87.500 |
| *Nocardia* | 0 | 26.90 | 6.00 | 7.90 | 49.01 | 74.3 | 0.555 | 47.772 | 8.298 | 39.710 | 87.500 |
| *Nocardioides* | 0 | 26.90 | 6.00 | 7.90 | 49.01 | 74.3 | 0.555 | 47.772 | 8.298 | 39.710 | 87.500 |
| *proteobacteria* | 1 | 26.90 | 6.00 | 7.90 | 49.01 | 74.3 | 0.555 | 47.772 | 8.298 | 39.710 | 87.500 |
| *Pseudomonas* | 0 | 26.90 | 6.00 | 7.90 | 49.01 | 74.3 | 0.555 | 47.772 | 8.298 | 39.710 | 87.500 |
| *Rhodococcus* | 0 | 26.90 | 6.00 | 7.90 | 49.01 | 74.3 | 0.555 | 47.772 | 8.298 | 39.710 | 87.500 |
| *Sphingomonas* | 1 | 26.90 | 6.00 | 7.90 | 49.01 | 74.3 | 0.555 | 47.772 | 8.298 | 39.710 | 87.500 |
| *Staphylococcus* | 0 | 26.90 | 6.00 | 7.90 | 49.01 | 74.3 | 0.555 | 47.772 | 8.298 | 39.710 | 87.500 |
| *Stenotrophomonas* | 0 | 26.90 | 6.00 | 7.90 | 49.01 | 74.3 | 0.555 | 47.772 | 8.298 | 39.710 | 87.500 |
| *Streptomyces* | 0 | 26.90 | 6.00 | 7.90 | 49.01 | 74.3 | 0.555 | 47.772 | 8.298 | 39.710 | 87.500 |
| *Terrabacter* | 0 | 26.90 | 6.00 | 7.90 | 49.01 | 74.3 | 0.555 | 47.772 | 8.298 | 39.710 | 87.500 |
| *Variovorax* | 0 | 26.90 | 6.00 | 7.90 | 49.01 | 74.3 | 0.555 | 47.772 | 8.298 | 39.710 | 87.500 |
| *Verrucosispora* | 0 | 26.90 | 6.00 | 7.90 | 49.01 | 74.3 | 0.555 | 47.772 | 8.298 | 39.710 | 87.500 |
| *Agrococcus* | 0 | 28.20 | 2.40 | 7.90 | 31.76 | 73.1 | 0.000 | 160.141 | 5.126 | 22.850 | 57.100 |
| *Alcaligenes* | 0 | 28.20 | 2.40 | 7.90 | 31.76 | 73.1 | 0.000 | 160.141 | 5.126 | 22.850 | 57.100 |
| *Arthrobacter* | 1 | 28.20 | 2.40 | 7.90 | 31.76 | 73.1 | 0.000 | 160.141 | 5.126 | 22.850 | 57.100 |
| *Azospirillum* | 0 | 28.20 | 2.40 | 7.90 | 31.76 | 73.1 | 0.000 | 160.141 | 5.126 | 22.850 | 57.100 |
| *Bacillus* | 0 | 28.20 | 2.40 | 7.90 | 31.76 | 73.1 | 0.000 | 160.141 | 5.126 | 22.850 | 57.100 |
| *Bradyrhizobium* | 0 | 28.20 | 2.40 | 7.90 | 31.76 | 73.1 | 0.000 | 160.141 | 5.126 | 22.850 | 57.100 |
| *Clavibacter* | 0 | 28.20 | 2.40 | 7.90 | 31.76 | 73.1 | 0.000 | 160.141 | 5.126 | 22.850 | 57.100 |
| *Deinococcus* | 0 | 28.20 | 2.40 | 7.90 | 31.76 | 73.1 | 0.000 | 160.141 | 5.126 | 22.850 | 57.100 |
| *Dermabacter* | 1 | 28.20 | 2.40 | 7.90 | 31.76 | 73.1 | 0.000 | 160.141 | 5.126 | 22.850 | 57.100 |
| *Detolaasinbacter* | 0 | 28.20 | 2.40 | 7.90 | 31.76 | 73.1 | 0.000 | 160.141 | 5.126 | 22.850 | 57.100 |
| *Janibacter* | 0 | 28.20 | 2.40 | 7.90 | 31.76 | 73.1 | 0.000 | 160.141 | 5.126 | 22.850 | 57.100 |
| *Microbacterium* | 0 | 28.20 | 2.40 | 7.90 | 31.76 | 73.1 | 0.000 | 160.141 | 5.126 | 22.850 | 57.100 |
| *Micrococcus* | 0 | 28.20 | 2.40 | 7.90 | 31.76 | 73.1 | 0.000 | 160.141 | 5.126 | 22.850 | 57.100 |
| *Mycobacterium* | 0 | 28.20 | 2.40 | 7.90 | 31.76 | 73.1 | 0.000 | 160.141 | 5.126 | 22.850 | 57.100 |
| *Nocardia* | 0 | 28.20 | 2.40 | 7.90 | 31.76 | 73.1 | 0.000 | 160.141 | 5.126 | 22.850 | 57.100 |
| *Nocardioides* | 0 | 28.20 | 2.40 | 7.90 | 31.76 | 73.1 | 0.000 | 160.141 | 5.126 | 22.850 | 57.100 |
| *proteobacteria* | 1 | 28.20 | 2.40 | 7.90 | 31.76 | 73.1 | 0.000 | 160.141 | 5.126 | 22.850 | 57.100 |
| *Pseudomonas* | 0 | 28.20 | 2.40 | 7.90 | 31.76 | 73.1 | 0.000 | 160.141 | 5.126 | 22.850 | 57.100 |
| *Rhodococcus* | 0 | 28.20 | 2.40 | 7.90 | 31.76 | 73.1 | 0.000 | 160.141 | 5.126 | 22.850 | 57.100 |
| *Sphingomonas* | 0 | 28.20 | 2.40 | 7.90 | 31.76 | 73.1 | 0.000 | 160.141 | 5.126 | 22.850 | 57.100 |
| *Staphylococcus* | 0 | 28.20 | 2.40 | 7.90 | 31.76 | 73.1 | 0.000 | 160.141 | 5.126 | 22.850 | 57.100 |
| *Stenotrophomonas* | 0 | 28.20 | 2.40 | 7.90 | 31.76 | 73.1 | 0.000 | 160.141 | 5.126 | 22.850 | 57.100 |
| *Streptomyces* | 0 | 28.20 | 2.40 | 7.90 | 31.76 | 73.1 | 0.000 | 160.141 | 5.126 | 22.850 | 57.100 |
| *Terrabacter* | 0 | 28.20 | 2.40 | 7.90 | 31.76 | 73.1 | 0.000 | 160.141 | 5.126 | 22.850 | 57.100 |
| *Variovorax* | 0 | 28.20 | 2.40 | 7.90 | 31.76 | 73.1 | 0.000 | 160.141 | 5.126 | 22.850 | 57.100 |
| *Verrucosispora* | 1 | 28.20 | 2.40 | 7.90 | 31.76 | 73.1 | 0.000 | 160.141 | 5.126 | 22.850 | 57.100 |
| *Agrococcus* | 0 | 29.50 | 1.90 | 8.20 | 25.56 | 71.5 | 0.000 | 226.254 | 3.981 | 18.990 | 59.000 |
| *Alcaligenes* | 0 | 29.50 | 1.90 | 8.20 | 25.56 | 71.5 | 0.000 | 226.254 | 3.981 | 18.990 | 59.000 |
| *Arthrobacter* | 0 | 29.50 | 1.90 | 8.20 | 25.56 | 71.5 | 0.000 | 226.254 | 3.981 | 18.990 | 59.000 |
| *Azospirillum* | 0 | 29.50 | 1.90 | 8.20 | 25.56 | 71.5 | 0.000 | 226.254 | 3.981 | 18.990 | 59.000 |
| *Bacillus* | 0 | 29.50 | 1.90 | 8.20 | 25.56 | 71.5 | 0.000 | 226.254 | 3.981 | 18.990 | 59.000 |
| *Bradyrhizobium* | 0 | 29.50 | 1.90 | 8.20 | 25.56 | 71.5 | 0.000 | 226.254 | 3.981 | 18.990 | 59.000 |
| *Clavibacter* | 0 | 29.50 | 1.90 | 8.20 | 25.56 | 71.5 | 0.000 | 226.254 | 3.981 | 18.990 | 59.000 |
| *Deinococcus* | 0 | 29.50 | 1.90 | 8.20 | 25.56 | 71.5 | 0.000 | 226.254 | 3.981 | 18.990 | 59.000 |
| *Dermabacter* | 0 | 29.50 | 1.90 | 8.20 | 25.56 | 71.5 | 0.000 | 226.254 | 3.981 | 18.990 | 59.000 |
| *Detolaasinbacter* | 0 | 29.50 | 1.90 | 8.20 | 25.56 | 71.5 | 0.000 | 226.254 | 3.981 | 18.990 | 59.000 |
| *Janibacter* | 0 | 29.50 | 1.90 | 8.20 | 25.56 | 71.5 | 0.000 | 226.254 | 3.981 | 18.990 | 59.000 |
| *Microbacterium* | 0 | 29.50 | 1.90 | 8.20 | 25.56 | 71.5 | 0.000 | 226.254 | 3.981 | 18.990 | 59.000 |
| *Micrococcus* | 0 | 29.50 | 1.90 | 8.20 | 25.56 | 71.5 | 0.000 | 226.254 | 3.981 | 18.990 | 59.000 |
| *Mycobacterium* | 0 | 29.50 | 1.90 | 8.20 | 25.56 | 71.5 | 0.000 | 226.254 | 3.981 | 18.990 | 59.000 |
| *Nocardia* | 0 | 29.50 | 1.90 | 8.20 | 25.56 | 71.5 | 0.000 | 226.254 | 3.981 | 18.990 | 59.000 |
| *Nocardioides* | 0 | 29.50 | 1.90 | 8.20 | 25.56 | 71.5 | 0.000 | 226.254 | 3.981 | 18.990 | 59.000 |
| *proteobacteria* | 1 | 29.50 | 1.90 | 8.20 | 25.56 | 71.5 | 0.000 | 226.254 | 3.981 | 18.990 | 59.000 |
| *Pseudomonas* | 0 | 29.50 | 1.90 | 8.20 | 25.56 | 71.5 | 0.000 | 226.254 | 3.981 | 18.990 | 59.000 |
| *Rhodococcus* | 0 | 29.50 | 1.90 | 8.20 | 25.56 | 71.5 | 0.000 | 226.254 | 3.981 | 18.990 | 59.000 |
| *Sphingomonas* | 0 | 29.50 | 1.90 | 8.20 | 25.56 | 71.5 | 0.000 | 226.254 | 3.981 | 18.990 | 59.000 |
| *Staphylococcus* | 0 | 29.50 | 1.90 | 8.20 | 25.56 | 71.5 | 0.000 | 226.254 | 3.981 | 18.990 | 59.000 |
| *Stenotrophomonas* | 0 | 29.50 | 1.90 | 8.20 | 25.56 | 71.5 | 0.000 | 226.254 | 3.981 | 18.990 | 59.000 |
| *Streptomyces* | 0 | 29.50 | 1.90 | 8.20 | 25.56 | 71.5 | 0.000 | 226.254 | 3.981 | 18.990 | 59.000 |
| *Terrabacter* | 0 | 29.50 | 1.90 | 8.20 | 25.56 | 71.5 | 0.000 | 226.254 | 3.981 | 18.990 | 59.000 |
| *Variovorax* | 0 | 29.50 | 1.90 | 8.20 | 25.56 | 71.5 | 0.000 | 226.254 | 3.981 | 18.990 | 59.000 |
| *Verrucosispora* | 0 | 29.50 | 1.90 | 8.20 | 25.56 | 71.5 | 0.000 | 226.254 | 3.981 | 18.990 | 59.000 |
| *Agrococcus* | 0 | 30.80 | 3.20 | 8.40 | 13.93 | 72.5 | 0.001 | 134.727 | 0.009 | 9.520 | 5.000 |
| *Alcaligenes* | 0 | 30.80 | 3.20 | 8.40 | 13.93 | 72.5 | 0.001 | 134.727 | 0.009 | 9.520 | 5.000 |
| *Arthrobacter* | 0 | 30.80 | 3.20 | 8.40 | 13.93 | 72.5 | 0.001 | 134.727 | 0.009 | 9.520 | 5.000 |
| *Azospirillum* | 0 | 30.80 | 3.20 | 8.40 | 13.93 | 72.5 | 0.001 | 134.727 | 0.009 | 9.520 | 5.000 |
| *Bacillus* | 0 | 30.80 | 3.20 | 8.40 | 13.93 | 72.5 | 0.001 | 134.727 | 0.009 | 9.520 | 5.000 |
| *Bradyrhizobium* | 0 | 30.80 | 3.20 | 8.40 | 13.93 | 72.5 | 0.001 | 134.727 | 0.009 | 9.520 | 5.000 |
| *Clavibacter* | 0 | 30.80 | 3.20 | 8.40 | 13.93 | 72.5 | 0.001 | 134.727 | 0.009 | 9.520 | 5.000 |
| *Deinococcus* | 0 | 30.80 | 3.20 | 8.40 | 13.93 | 72.5 | 0.001 | 134.727 | 0.009 | 9.520 | 5.000 |
| *Dermabacter* | 0 | 30.80 | 3.20 | 8.40 | 13.93 | 72.5 | 0.001 | 134.727 | 0.009 | 9.520 | 5.000 |
| *Detolaasinbacter* | 0 | 30.80 | 3.20 | 8.40 | 13.93 | 72.5 | 0.001 | 134.727 | 0.009 | 9.520 | 5.000 |
| *Janibacter* | 0 | 30.80 | 3.20 | 8.40 | 13.93 | 72.5 | 0.001 | 134.727 | 0.009 | 9.520 | 5.000 |
| *Microbacterium* | 0 | 30.80 | 3.20 | 8.40 | 13.93 | 72.5 | 0.001 | 134.727 | 0.009 | 9.520 | 5.000 |
| *Micrococcus* | 0 | 30.80 | 3.20 | 8.40 | 13.93 | 72.5 | 0.001 | 134.727 | 0.009 | 9.520 | 5.000 |
| *Mycobacterium* | 0 | 30.80 | 3.20 | 8.40 | 13.93 | 72.5 | 0.001 | 134.727 | 0.009 | 9.520 | 5.000 |
| *Nocardia* | 0 | 30.80 | 3.20 | 8.40 | 13.93 | 72.5 | 0.001 | 134.727 | 0.009 | 9.520 | 5.000 |
| *Nocardioides* | 0 | 30.80 | 3.20 | 8.40 | 13.93 | 72.5 | 0.001 | 134.727 | 0.009 | 9.520 | 5.000 |
| *proteobacteria* | 0 | 30.80 | 3.20 | 8.40 | 13.93 | 72.5 | 0.001 | 134.727 | 0.009 | 9.520 | 5.000 |
| *Pseudomonas* | 0 | 30.80 | 3.20 | 8.40 | 13.93 | 72.5 | 0.001 | 134.727 | 0.009 | 9.520 | 5.000 |
| *Rhodococcus* | 0 | 30.80 | 3.20 | 8.40 | 13.93 | 72.5 | 0.001 | 134.727 | 0.009 | 9.520 | 5.000 |
| *Sphingomonas* | 0 | 30.80 | 3.20 | 8.40 | 13.93 | 72.5 | 0.001 | 134.727 | 0.009 | 9.520 | 5.000 |
| *Staphylococcus* | 0 | 30.80 | 3.20 | 8.40 | 13.93 | 72.5 | 0.001 | 134.727 | 0.009 | 9.520 | 5.000 |
| *Stenotrophomonas* | 0 | 30.80 | 3.20 | 8.40 | 13.93 | 72.5 | 0.001 | 134.727 | 0.009 | 9.520 | 5.000 |
| *Streptomyces* | 0 | 30.80 | 3.20 | 8.40 | 13.93 | 72.5 | 0.001 | 134.727 | 0.009 | 9.520 | 5.000 |
| *Terrabacter* | 0 | 30.80 | 3.20 | 8.40 | 13.93 | 72.5 | 0.001 | 134.727 | 0.009 | 9.520 | 5.000 |
| *Variovorax* | 0 | 30.80 | 3.20 | 8.40 | 13.93 | 72.5 | 0.001 | 134.727 | 0.009 | 9.520 | 5.000 |
| *Verrucosispora* | 0 | 30.80 | 3.20 | 8.40 | 13.93 | 72.5 | 0.001 | 134.727 | 0.009 | 9.520 | 5.000 |
| *Agrococcus* | 0 | 32.00 | 21.40 | 8.00 | 2.36 | 72.2 | 0.000 | 2.531 | 0.003 | 1.530 | 0.500 |
| *Alcaligenes* | 0 | 32.00 | 21.40 | 8.00 | 2.36 | 72.2 | 0.000 | 2.531 | 0.003 | 1.530 | 0.500 |
| *Arthrobacter* | 0 | 32.00 | 21.40 | 8.00 | 2.36 | 72.2 | 0.000 | 2.531 | 0.003 | 1.530 | 0.500 |
| *Azospirillum* | 0 | 32.00 | 21.40 | 8.00 | 2.36 | 72.2 | 0.000 | 2.531 | 0.003 | 1.530 | 0.500 |
| *Bacillus* | 0 | 32.00 | 21.40 | 8.00 | 2.36 | 72.2 | 0.000 | 2.531 | 0.003 | 1.530 | 0.500 |
| *Bradyrhizobium* | 0 | 32.00 | 21.40 | 8.00 | 2.36 | 72.2 | 0.000 | 2.531 | 0.003 | 1.530 | 0.500 |
| *Clavibacter* | 0 | 32.00 | 21.40 | 8.00 | 2.36 | 72.2 | 0.000 | 2.531 | 0.003 | 1.530 | 0.500 |
| *Deinococcus* | 0 | 32.00 | 21.40 | 8.00 | 2.36 | 72.2 | 0.000 | 2.531 | 0.003 | 1.530 | 0.500 |
| *Dermabacter* | 0 | 32.00 | 21.40 | 8.00 | 2.36 | 72.2 | 0.000 | 2.531 | 0.003 | 1.530 | 0.500 |
| *Detolaasinbacter* | 0 | 32.00 | 21.40 | 8.00 | 2.36 | 72.2 | 0.000 | 2.531 | 0.003 | 1.530 | 0.500 |
| *Janibacter* | 0 | 32.00 | 21.40 | 8.00 | 2.36 | 72.2 | 0.000 | 2.531 | 0.003 | 1.530 | 0.500 |
| *Microbacterium* | 0 | 32.00 | 21.40 | 8.00 | 2.36 | 72.2 | 0.000 | 2.531 | 0.003 | 1.530 | 0.500 |
| *Micrococcus* | 0 | 32.00 | 21.40 | 8.00 | 2.36 | 72.2 | 0.000 | 2.531 | 0.003 | 1.530 | 0.500 |
| *Mycobacterium* | 0 | 32.00 | 21.40 | 8.00 | 2.36 | 72.2 | 0.000 | 2.531 | 0.003 | 1.530 | 0.500 |
| *Nocardia* | 0 | 32.00 | 21.40 | 8.00 | 2.36 | 72.2 | 0.000 | 2.531 | 0.003 | 1.530 | 0.500 |
| *Nocardioides* | 0 | 32.00 | 21.40 | 8.00 | 2.36 | 72.2 | 0.000 | 2.531 | 0.003 | 1.530 | 0.500 |
| *proteobacteria* | 0 | 32.00 | 21.40 | 8.00 | 2.36 | 72.2 | 0.000 | 2.531 | 0.003 | 1.530 | 0.500 |
| *Pseudomonas* | 1 | 32.00 | 21.40 | 8.00 | 2.36 | 72.2 | 0.000 | 2.531 | 0.003 | 1.530 | 0.500 |
| *Rhodococcus* | 0 | 32.00 | 21.40 | 8.00 | 2.36 | 72.2 | 0.000 | 2.531 | 0.003 | 1.530 | 0.500 |
| *Sphingomonas* | 0 | 32.00 | 21.40 | 8.00 | 2.36 | 72.2 | 0.000 | 2.531 | 0.003 | 1.530 | 0.500 |
| *Staphylococcus* | 0 | 32.00 | 21.40 | 8.00 | 2.36 | 72.2 | 0.000 | 2.531 | 0.003 | 1.530 | 0.500 |
| *Stenotrophomonas* | 0 | 32.00 | 21.40 | 8.00 | 2.36 | 72.2 | 0.000 | 2.531 | 0.003 | 1.530 | 0.500 |
| *Streptomyces* | 0 | 32.00 | 21.40 | 8.00 | 2.36 | 72.2 | 0.000 | 2.531 | 0.003 | 1.530 | 0.500 |
| *Terrabacter* | 0 | 32.00 | 21.40 | 8.00 | 2.36 | 72.2 | 0.000 | 2.531 | 0.003 | 1.530 | 0.500 |
| *Variovorax* | 0 | 32.00 | 21.40 | 8.00 | 2.36 | 72.2 | 0.000 | 2.531 | 0.003 | 1.530 | 0.500 |
| *Verrucosispora* | 0 | 32.00 | 21.40 | 8.00 | 2.36 | 72.2 | 0.000 | 2.531 | 0.003 | 1.530 | 0.500 |
| *Agrococcus* | 0 | 34.50 | 7.60 | 8.00 | 29.78 | 68.9 | 0.001 | 95.359 | 4.310 | 22.200 | 72.500 |
| *Alcaligenes* | 0 | 34.50 | 7.60 | 8.00 | 29.78 | 68.9 | 0.001 | 95.359 | 4.310 | 22.200 | 72.500 |
| *Arthrobacter* | 0 | 34.50 | 7.60 | 8.00 | 29.78 | 68.9 | 0.001 | 95.359 | 4.310 | 22.200 | 72.500 |
| *Azospirillum* | 0 | 34.50 | 7.60 | 8.00 | 29.78 | 68.9 | 0.001 | 95.359 | 4.310 | 22.200 | 72.500 |
| *Bacillus* | 0 | 34.50 | 7.60 | 8.00 | 29.78 | 68.9 | 0.001 | 95.359 | 4.310 | 22.200 | 72.500 |
| *Bradyrhizobium* | 0 | 34.50 | 7.60 | 8.00 | 29.78 | 68.9 | 0.001 | 95.359 | 4.310 | 22.200 | 72.500 |
| *Clavibacter* | 0 | 34.50 | 7.60 | 8.00 | 29.78 | 68.9 | 0.001 | 95.359 | 4.310 | 22.200 | 72.500 |
| *Deinococcus* | 0 | 34.50 | 7.60 | 8.00 | 29.78 | 68.9 | 0.001 | 95.359 | 4.310 | 22.200 | 72.500 |
| *Dermabacter* | 0 | 34.50 | 7.60 | 8.00 | 29.78 | 68.9 | 0.001 | 95.359 | 4.310 | 22.200 | 72.500 |
| *Detolaasinbacter* | 0 | 34.50 | 7.60 | 8.00 | 29.78 | 68.9 | 0.001 | 95.359 | 4.310 | 22.200 | 72.500 |
| *Janibacter* | 0 | 34.50 | 7.60 | 8.00 | 29.78 | 68.9 | 0.001 | 95.359 | 4.310 | 22.200 | 72.500 |
| *Microbacterium* | 0 | 34.50 | 7.60 | 8.00 | 29.78 | 68.9 | 0.001 | 95.359 | 4.310 | 22.200 | 72.500 |
| *Micrococcus* | 0 | 34.50 | 7.60 | 8.00 | 29.78 | 68.9 | 0.001 | 95.359 | 4.310 | 22.200 | 72.500 |
| *Mycobacterium* | 0 | 34.50 | 7.60 | 8.00 | 29.78 | 68.9 | 0.001 | 95.359 | 4.310 | 22.200 | 72.500 |
| *Nocardia* | 0 | 34.50 | 7.60 | 8.00 | 29.78 | 68.9 | 0.001 | 95.359 | 4.310 | 22.200 | 72.500 |
| *Nocardioides* | 0 | 34.50 | 7.60 | 8.00 | 29.78 | 68.9 | 0.001 | 95.359 | 4.310 | 22.200 | 72.500 |
| *proteobacteria* | 0 | 34.50 | 7.60 | 8.00 | 29.78 | 68.9 | 0.001 | 95.359 | 4.310 | 22.200 | 72.500 |
| *Pseudomonas* | 0 | 34.50 | 7.60 | 8.00 | 29.78 | 68.9 | 0.001 | 95.359 | 4.310 | 22.200 | 72.500 |
| *Rhodococcus* | 0 | 34.50 | 7.60 | 8.00 | 29.78 | 68.9 | 0.001 | 95.359 | 4.310 | 22.200 | 72.500 |
| *Sphingomonas* | 0 | 34.50 | 7.60 | 8.00 | 29.78 | 68.9 | 0.001 | 95.359 | 4.310 | 22.200 | 72.500 |
| *Staphylococcus* | 0 | 34.50 | 7.60 | 8.00 | 29.78 | 68.9 | 0.001 | 95.359 | 4.310 | 22.200 | 72.500 |
| *Stenotrophomonas* | 0 | 34.50 | 7.60 | 8.00 | 29.78 | 68.9 | 0.001 | 95.359 | 4.310 | 22.200 | 72.500 |
| *Streptomyces* | 0 | 34.50 | 7.60 | 8.00 | 29.78 | 68.9 | 0.001 | 95.359 | 4.310 | 22.200 | 72.500 |
| *Terrabacter* | 0 | 34.50 | 7.60 | 8.00 | 29.78 | 68.9 | 0.001 | 95.359 | 4.310 | 22.200 | 72.500 |
| *Variovorax* | 0 | 34.50 | 7.60 | 8.00 | 29.78 | 68.9 | 0.001 | 95.359 | 4.310 | 22.200 | 72.500 |
| *Verrucosispora* | 0 | 34.50 | 7.60 | 8.00 | 29.78 | 68.9 | 0.001 | 95.359 | 4.310 | 22.200 | 72.500 |
| *Agrococcus* | 0 | 37.00 | 12.00 | 7.80 | 30.24 | 67.1 | 0.001 | 56.783 | 2.978 | 21.500 | 46.300 |
| *Alcaligenes* | 0 | 37.00 | 12.00 | 7.80 | 30.24 | 67.1 | 0.001 | 56.783 | 2.978 | 21.500 | 46.300 |
| *Arthrobacter* | 0 | 37.00 | 12.00 | 7.80 | 30.24 | 67.1 | 0.001 | 56.783 | 2.978 | 21.500 | 46.300 |
| *Azospirillum* | 0 | 37.00 | 12.00 | 7.80 | 30.24 | 67.1 | 0.001 | 56.783 | 2.978 | 21.500 | 46.300 |
| *Bacillus* | 0 | 37.00 | 12.00 | 7.80 | 30.24 | 67.1 | 0.001 | 56.783 | 2.978 | 21.500 | 46.300 |
| *Bradyrhizobium* | 0 | 37.00 | 12.00 | 7.80 | 30.24 | 67.1 | 0.001 | 56.783 | 2.978 | 21.500 | 46.300 |
| *Clavibacter* | 0 | 37.00 | 12.00 | 7.80 | 30.24 | 67.1 | 0.001 | 56.783 | 2.978 | 21.500 | 46.300 |
| *Deinococcus* | 0 | 37.00 | 12.00 | 7.80 | 30.24 | 67.1 | 0.001 | 56.783 | 2.978 | 21.500 | 46.300 |
| *Dermabacter* | 0 | 37.00 | 12.00 | 7.80 | 30.24 | 67.1 | 0.001 | 56.783 | 2.978 | 21.500 | 46.300 |
| *Detolaasinbacter* | 0 | 37.00 | 12.00 | 7.80 | 30.24 | 67.1 | 0.001 | 56.783 | 2.978 | 21.500 | 46.300 |
| *Janibacter* | 0 | 37.00 | 12.00 | 7.80 | 30.24 | 67.1 | 0.001 | 56.783 | 2.978 | 21.500 | 46.300 |
| *Microbacterium* | 0 | 37.00 | 12.00 | 7.80 | 30.24 | 67.1 | 0.001 | 56.783 | 2.978 | 21.500 | 46.300 |
| *Micrococcus* | 0 | 37.00 | 12.00 | 7.80 | 30.24 | 67.1 | 0.001 | 56.783 | 2.978 | 21.500 | 46.300 |
| *Mycobacterium* | 0 | 37.00 | 12.00 | 7.80 | 30.24 | 67.1 | 0.001 | 56.783 | 2.978 | 21.500 | 46.300 |
| *Nocardia* | 0 | 37.00 | 12.00 | 7.80 | 30.24 | 67.1 | 0.001 | 56.783 | 2.978 | 21.500 | 46.300 |
| *Nocardioides* | 0 | 37.00 | 12.00 | 7.80 | 30.24 | 67.1 | 0.001 | 56.783 | 2.978 | 21.500 | 46.300 |
| *proteobacteria* | 0 | 37.00 | 12.00 | 7.80 | 30.24 | 67.1 | 0.001 | 56.783 | 2.978 | 21.500 | 46.300 |
| *Pseudomonas* | 0 | 37.00 | 12.00 | 7.80 | 30.24 | 67.1 | 0.001 | 56.783 | 2.978 | 21.500 | 46.300 |
| *Rhodococcus* | 0 | 37.00 | 12.00 | 7.80 | 30.24 | 67.1 | 0.001 | 56.783 | 2.978 | 21.500 | 46.300 |
| *Sphingomonas* | 0 | 37.00 | 12.00 | 7.80 | 30.24 | 67.1 | 0.001 | 56.783 | 2.978 | 21.500 | 46.300 |
| *Staphylococcus* | 0 | 37.00 | 12.00 | 7.80 | 30.24 | 67.1 | 0.001 | 56.783 | 2.978 | 21.500 | 46.300 |
| *Stenotrophomonas* | 0 | 37.00 | 12.00 | 7.80 | 30.24 | 67.1 | 0.001 | 56.783 | 2.978 | 21.500 | 46.300 |
| *Streptomyces* | 0 | 37.00 | 12.00 | 7.80 | 30.24 | 67.1 | 0.001 | 56.783 | 2.978 | 21.500 | 46.300 |
| *Terrabacter* | 0 | 37.00 | 12.00 | 7.80 | 30.24 | 67.1 | 0.001 | 56.783 | 2.978 | 21.500 | 46.300 |
| *Variovorax* | 0 | 37.00 | 12.00 | 7.80 | 30.24 | 67.1 | 0.001 | 56.783 | 2.978 | 21.500 | 46.300 |
| *Verrucosispora* | 0 | 37.00 | 12.00 | 7.80 | 30.24 | 67.1 | 0.001 | 56.783 | 2.978 | 21.500 | 46.300 |
| *Agrococcus* | 0 | 39.50 | 17.40 | 7.50 | 40.01 | 63.8 | 0.001 | 67.494 | 3.365 | 34.600 | 34.400 |
| *Alcaligenes* | 0 | 39.50 | 17.40 | 7.50 | 40.01 | 63.8 | 0.001 | 67.494 | 3.365 | 34.600 | 34.400 |
| *Arthrobacter* | 0 | 39.50 | 17.40 | 7.50 | 40.01 | 63.8 | 0.001 | 67.494 | 3.365 | 34.600 | 34.400 |
| *Azospirillum* | 1 | 39.50 | 17.40 | 7.50 | 40.01 | 63.8 | 0.001 | 67.494 | 3.365 | 34.600 | 34.400 |
| *Bacillus* | 0 | 39.50 | 17.40 | 7.50 | 40.01 | 63.8 | 0.001 | 67.494 | 3.365 | 34.600 | 34.400 |
| *Bradyrhizobium* | 0 | 39.50 | 17.40 | 7.50 | 40.01 | 63.8 | 0.001 | 67.494 | 3.365 | 34.600 | 34.400 |
| *Clavibacter* | 0 | 39.50 | 17.40 | 7.50 | 40.01 | 63.8 | 0.001 | 67.494 | 3.365 | 34.600 | 34.400 |
| *Deinococcus* | 0 | 39.50 | 17.40 | 7.50 | 40.01 | 63.8 | 0.001 | 67.494 | 3.365 | 34.600 | 34.400 |
| *Dermabacter* | 0 | 39.50 | 17.40 | 7.50 | 40.01 | 63.8 | 0.001 | 67.494 | 3.365 | 34.600 | 34.400 |
| *Detolaasinbacter* | 0 | 39.50 | 17.40 | 7.50 | 40.01 | 63.8 | 0.001 | 67.494 | 3.365 | 34.600 | 34.400 |
| *Janibacter* | 0 | 39.50 | 17.40 | 7.50 | 40.01 | 63.8 | 0.001 | 67.494 | 3.365 | 34.600 | 34.400 |
| *Microbacterium* | 0 | 39.50 | 17.40 | 7.50 | 40.01 | 63.8 | 0.001 | 67.494 | 3.365 | 34.600 | 34.400 |
| *Micrococcus* | 0 | 39.50 | 17.40 | 7.50 | 40.01 | 63.8 | 0.001 | 67.494 | 3.365 | 34.600 | 34.400 |
| *Mycobacterium* | 0 | 39.50 | 17.40 | 7.50 | 40.01 | 63.8 | 0.001 | 67.494 | 3.365 | 34.600 | 34.400 |
| *Nocardia* | 0 | 39.50 | 17.40 | 7.50 | 40.01 | 63.8 | 0.001 | 67.494 | 3.365 | 34.600 | 34.400 |
| *Nocardioides* | 0 | 39.50 | 17.40 | 7.50 | 40.01 | 63.8 | 0.001 | 67.494 | 3.365 | 34.600 | 34.400 |
| *proteobacteria* | 0 | 39.50 | 17.40 | 7.50 | 40.01 | 63.8 | 0.001 | 67.494 | 3.365 | 34.600 | 34.400 |
| *Pseudomonas* | 0 | 39.50 | 17.40 | 7.50 | 40.01 | 63.8 | 0.001 | 67.494 | 3.365 | 34.600 | 34.400 |
| *Rhodococcus* | 0 | 39.50 | 17.40 | 7.50 | 40.01 | 63.8 | 0.001 | 67.494 | 3.365 | 34.600 | 34.400 |
| *Sphingomonas* | 0 | 39.50 | 17.40 | 7.50 | 40.01 | 63.8 | 0.001 | 67.494 | 3.365 | 34.600 | 34.400 |
| *Staphylococcus* | 0 | 39.50 | 17.40 | 7.50 | 40.01 | 63.8 | 0.001 | 67.494 | 3.365 | 34.600 | 34.400 |
| *Stenotrophomonas* | 0 | 39.50 | 17.40 | 7.50 | 40.01 | 63.8 | 0.001 | 67.494 | 3.365 | 34.600 | 34.400 |
| *Streptomyces* | 0 | 39.50 | 17.40 | 7.50 | 40.01 | 63.8 | 0.001 | 67.494 | 3.365 | 34.600 | 34.400 |
| *Terrabacter* | 1 | 39.50 | 17.40 | 7.50 | 40.01 | 63.8 | 0.001 | 67.494 | 3.365 | 34.600 | 34.400 |
| *Variovorax* | 0 | 39.50 | 17.40 | 7.50 | 40.01 | 63.8 | 0.001 | 67.494 | 3.365 | 34.600 | 34.400 |
| *Verrucosispora* | 0 | 39.50 | 17.40 | 7.50 | 40.01 | 63.8 | 0.001 | 67.494 | 3.365 | 34.600 | 34.400 |
| *Agrococcus* | 0 | 41.90 | 7.50 | 7.20 | 5.80 | 60.5 | 0.000 | 18.790 | 0.001 | 4.190 | 5.000 |
| *Alcaligenes* | 0 | 41.90 | 7.50 | 7.20 | 5.80 | 60.5 | 0.000 | 18.790 | 0.001 | 4.190 | 5.000 |
| *Arthrobacter* | 1 | 41.90 | 7.50 | 7.20 | 5.80 | 60.5 | 0.000 | 18.790 | 0.001 | 4.190 | 5.000 |
| *Azospirillum* | 0 | 41.90 | 7.50 | 7.20 | 5.80 | 60.5 | 0.000 | 18.790 | 0.001 | 4.190 | 5.000 |
| *Bacillus* | 0 | 41.90 | 7.50 | 7.20 | 5.80 | 60.5 | 0.000 | 18.790 | 0.001 | 4.190 | 5.000 |
| *Bradyrhizobium* | 0 | 41.90 | 7.50 | 7.20 | 5.80 | 60.5 | 0.000 | 18.790 | 0.001 | 4.190 | 5.000 |
| *Clavibacter* | 0 | 41.90 | 7.50 | 7.20 | 5.80 | 60.5 | 0.000 | 18.790 | 0.001 | 4.190 | 5.000 |
| *Deinococcus* | 0 | 41.90 | 7.50 | 7.20 | 5.80 | 60.5 | 0.000 | 18.790 | 0.001 | 4.190 | 5.000 |
| *Dermabacter* | 0 | 41.90 | 7.50 | 7.20 | 5.80 | 60.5 | 0.000 | 18.790 | 0.001 | 4.190 | 5.000 |
| *Detolaasinbacter* | 0 | 41.90 | 7.50 | 7.20 | 5.80 | 60.5 | 0.000 | 18.790 | 0.001 | 4.190 | 5.000 |
| *Janibacter* | 0 | 41.90 | 7.50 | 7.20 | 5.80 | 60.5 | 0.000 | 18.790 | 0.001 | 4.190 | 5.000 |
| *Microbacterium* | 0 | 41.90 | 7.50 | 7.20 | 5.80 | 60.5 | 0.000 | 18.790 | 0.001 | 4.190 | 5.000 |
| *Micrococcus* | 0 | 41.90 | 7.50 | 7.20 | 5.80 | 60.5 | 0.000 | 18.790 | 0.001 | 4.190 | 5.000 |
| *Mycobacterium* | 0 | 41.90 | 7.50 | 7.20 | 5.80 | 60.5 | 0.000 | 18.790 | 0.001 | 4.190 | 5.000 |
| *Nocardia* | 0 | 41.90 | 7.50 | 7.20 | 5.80 | 60.5 | 0.000 | 18.790 | 0.001 | 4.190 | 5.000 |
| *Nocardioides* | 0 | 41.90 | 7.50 | 7.20 | 5.80 | 60.5 | 0.000 | 18.790 | 0.001 | 4.190 | 5.000 |
| *proteobacteria* | 0 | 41.90 | 7.50 | 7.20 | 5.80 | 60.5 | 0.000 | 18.790 | 0.001 | 4.190 | 5.000 |
| *Pseudomonas* | 0 | 41.90 | 7.50 | 7.20 | 5.80 | 60.5 | 0.000 | 18.790 | 0.001 | 4.190 | 5.000 |
| *Rhodococcus* | 0 | 41.90 | 7.50 | 7.20 | 5.80 | 60.5 | 0.000 | 18.790 | 0.001 | 4.190 | 5.000 |
| *Sphingomonas* | 0 | 41.90 | 7.50 | 7.20 | 5.80 | 60.5 | 0.000 | 18.790 | 0.001 | 4.190 | 5.000 |
| *Staphylococcus* | 1 | 41.90 | 7.50 | 7.20 | 5.80 | 60.5 | 0.000 | 18.790 | 0.001 | 4.190 | 5.000 |
| *Stenotrophomonas* | 0 | 41.90 | 7.50 | 7.20 | 5.80 | 60.5 | 0.000 | 18.790 | 0.001 | 4.190 | 5.000 |
| *Streptomyces* | 0 | 41.90 | 7.50 | 7.20 | 5.80 | 60.5 | 0.000 | 18.790 | 0.001 | 4.190 | 5.000 |
| *Terrabacter* | 0 | 41.90 | 7.50 | 7.20 | 5.80 | 60.5 | 0.000 | 18.790 | 0.001 | 4.190 | 5.000 |
| *Variovorax* | 0 | 41.90 | 7.50 | 7.20 | 5.80 | 60.5 | 0.000 | 18.790 | 0.001 | 4.190 | 5.000 |
| *Verrucosispora* | 0 | 41.90 | 7.50 | 7.20 | 5.80 | 60.5 | 0.000 | 18.790 | 0.001 | 4.190 | 5.000 |
| *Agrococcus* | 0 | 43.90 | 19.70 | 7.20 | 3.74 | 56.9 | 0.000 | 2.713 | 0.001 | 2.390 | 0.500 |
| *Alcaligenes* | 0 | 43.90 | 19.70 | 7.20 | 3.74 | 56.9 | 0.000 | 2.713 | 0.001 | 2.390 | 0.500 |
| *Arthrobacter* | 1 | 43.90 | 19.70 | 7.20 | 3.74 | 56.9 | 0.000 | 2.713 | 0.001 | 2.390 | 0.500 |
| *Azospirillum* | 0 | 43.90 | 19.70 | 7.20 | 3.74 | 56.9 | 0.000 | 2.713 | 0.001 | 2.390 | 0.500 |
| *Bacillus* | 0 | 43.90 | 19.70 | 7.20 | 3.74 | 56.9 | 0.000 | 2.713 | 0.001 | 2.390 | 0.500 |
| *Bradyrhizobium* | 0 | 43.90 | 19.70 | 7.20 | 3.74 | 56.9 | 0.000 | 2.713 | 0.001 | 2.390 | 0.500 |
| *Clavibacter* | 0 | 43.90 | 19.70 | 7.20 | 3.74 | 56.9 | 0.000 | 2.713 | 0.001 | 2.390 | 0.500 |
| *Deinococcus* | 0 | 43.90 | 19.70 | 7.20 | 3.74 | 56.9 | 0.000 | 2.713 | 0.001 | 2.390 | 0.500 |
| *Dermabacter* | 0 | 43.90 | 19.70 | 7.20 | 3.74 | 56.9 | 0.000 | 2.713 | 0.001 | 2.390 | 0.500 |
| *Detolaasinbacter* | 0 | 43.90 | 19.70 | 7.20 | 3.74 | 56.9 | 0.000 | 2.713 | 0.001 | 2.390 | 0.500 |
| *Janibacter* | 0 | 43.90 | 19.70 | 7.20 | 3.74 | 56.9 | 0.000 | 2.713 | 0.001 | 2.390 | 0.500 |
| *Microbacterium* | 0 | 43.90 | 19.70 | 7.20 | 3.74 | 56.9 | 0.000 | 2.713 | 0.001 | 2.390 | 0.500 |
| *Micrococcus* | 0 | 43.90 | 19.70 | 7.20 | 3.74 | 56.9 | 0.000 | 2.713 | 0.001 | 2.390 | 0.500 |
| *Mycobacterium* | 0 | 43.90 | 19.70 | 7.20 | 3.74 | 56.9 | 0.000 | 2.713 | 0.001 | 2.390 | 0.500 |
| *Nocardia* | 0 | 43.90 | 19.70 | 7.20 | 3.74 | 56.9 | 0.000 | 2.713 | 0.001 | 2.390 | 0.500 |
| *Nocardioides* | 0 | 43.90 | 19.70 | 7.20 | 3.74 | 56.9 | 0.000 | 2.713 | 0.001 | 2.390 | 0.500 |
| *proteobacteria* | 0 | 43.90 | 19.70 | 7.20 | 3.74 | 56.9 | 0.000 | 2.713 | 0.001 | 2.390 | 0.500 |
| *Pseudomonas* | 1 | 43.90 | 19.70 | 7.20 | 3.74 | 56.9 | 0.000 | 2.713 | 0.001 | 2.390 | 0.500 |
| *Rhodococcus* | 0 | 43.90 | 19.70 | 7.20 | 3.74 | 56.9 | 0.000 | 2.713 | 0.001 | 2.390 | 0.500 |
| *Sphingomonas* | 0 | 43.90 | 19.70 | 7.20 | 3.74 | 56.9 | 0.000 | 2.713 | 0.001 | 2.390 | 0.500 |
| *Staphylococcus* | 0 | 43.90 | 19.70 | 7.20 | 3.74 | 56.9 | 0.000 | 2.713 | 0.001 | 2.390 | 0.500 |
| *Stenotrophomonas* | 0 | 43.90 | 19.70 | 7.20 | 3.74 | 56.9 | 0.000 | 2.713 | 0.001 | 2.390 | 0.500 |
| *Streptomyces* | 1 | 43.90 | 19.70 | 7.20 | 3.74 | 56.9 | 0.000 | 2.713 | 0.001 | 2.390 | 0.500 |
| *Terrabacter* | 0 | 43.90 | 19.70 | 7.20 | 3.74 | 56.9 | 0.000 | 2.713 | 0.001 | 2.390 | 0.500 |
| *Variovorax* | 0 | 43.90 | 19.70 | 7.20 | 3.74 | 56.9 | 0.000 | 2.713 | 0.001 | 2.390 | 0.500 |
| *Verrucosispora* | 0 | 43.90 | 19.70 | 7.20 | 3.74 | 56.9 | 0.000 | 2.713 | 0.001 | 2.390 | 0.500 |
| *Agrococcus* | 0 | 25.00 | 4.70 | 9.60 | 54.62 | 40.0 | 0.000 | 0.000 | 0.000 | 0.000 | 0.000 |
| *Alcaligenes* | 1 | 25.00 | 4.70 | 9.60 | 54.62 | 40.0 | 0.000 | 0.000 | 0.000 | 0.000 | 0.000 |
| *Arthrobacter* | 1 | 25.00 | 4.70 | 9.60 | 54.62 | 40.0 | 0.000 | 0.000 | 0.000 | 0.000 | 0.000 |
| *Azospirillum* | 0 | 25.00 | 4.70 | 9.60 | 54.62 | 40.0 | 0.000 | 0.000 | 0.000 | 0.000 | 0.000 |
| *Bacillus* | 0 | 25.00 | 4.70 | 9.60 | 54.62 | 40.0 | 0.000 | 0.000 | 0.000 | 0.000 | 0.000 |
| *Bradyrhizobium* | 0 | 25.00 | 4.70 | 9.60 | 54.62 | 40.0 | 0.000 | 0.000 | 0.000 | 0.000 | 0.000 |
| *Clavibacter* | 0 | 25.00 | 4.70 | 9.60 | 54.62 | 40.0 | 0.000 | 0.000 | 0.000 | 0.000 | 0.000 |
| *Deinococcus* | 0 | 25.00 | 4.70 | 9.60 | 54.62 | 40.0 | 0.000 | 0.000 | 0.000 | 0.000 | 0.000 |
| *Dermabacter* | 0 | 25.00 | 4.70 | 9.60 | 54.62 | 40.0 | 0.000 | 0.000 | 0.000 | 0.000 | 0.000 |
| *Detolaasinbacter* | 0 | 25.00 | 4.70 | 9.60 | 54.62 | 40.0 | 0.000 | 0.000 | 0.000 | 0.000 | 0.000 |
| *Janibacter* | 1 | 25.00 | 4.70 | 9.60 | 54.62 | 40.0 | 0.000 | 0.000 | 0.000 | 0.000 | 0.000 |
| *Microbacterium* | 0 | 25.00 | 4.70 | 9.60 | 54.62 | 40.0 | 0.000 | 0.000 | 0.000 | 0.000 | 0.000 |
| *Micrococcus* | 0 | 25.00 | 4.70 | 9.60 | 54.62 | 40.0 | 0.000 | 0.000 | 0.000 | 0.000 | 0.000 |
| *Mycobacterium* | 1 | 25.00 | 4.70 | 9.60 | 54.62 | 40.0 | 0.000 | 0.000 | 0.000 | 0.000 | 0.000 |
| *Nocardia* | 0 | 25.00 | 4.70 | 9.60 | 54.62 | 40.0 | 0.000 | 0.000 | 0.000 | 0.000 | 0.000 |
| *Nocardioides* | 0 | 25.00 | 4.70 | 9.60 | 54.62 | 40.0 | 0.000 | 0.000 | 0.000 | 0.000 | 0.000 |
| *proteobacteria* | 0 | 25.00 | 4.70 | 9.60 | 54.62 | 40.0 | 0.000 | 0.000 | 0.000 | 0.000 | 0.000 |
| *Pseudomonas* | 0 | 25.00 | 4.70 | 9.60 | 54.62 | 40.0 | 0.000 | 0.000 | 0.000 | 0.000 | 0.000 |
| *Rhodococcus* | 0 | 25.00 | 4.70 | 9.60 | 54.62 | 40.0 | 0.000 | 0.000 | 0.000 | 0.000 | 0.000 |
| *Sphingomonas* | 0 | 25.00 | 4.70 | 9.60 | 54.62 | 40.0 | 0.000 | 0.000 | 0.000 | 0.000 | 0.000 |
| *Staphylococcus* | 0 | 25.00 | 4.70 | 9.60 | 54.62 | 40.0 | 0.000 | 0.000 | 0.000 | 0.000 | 0.000 |
| *Stenotrophomonas* | 0 | 25.00 | 4.70 | 9.60 | 54.62 | 40.0 | 0.000 | 0.000 | 0.000 | 0.000 | 0.000 |
| *Streptomyces* | 0 | 25.00 | 4.70 | 9.60 | 54.62 | 40.0 | 0.000 | 0.000 | 0.000 | 0.000 | 0.000 |
| *Terrabacter* | 1 | 25.00 | 4.70 | 9.60 | 54.62 | 40.0 | 0.000 | 0.000 | 0.000 | 0.000 | 0.000 |
| *Variovorax* | 1 | 25.00 | 4.70 | 9.60 | 54.62 | 40.0 | 0.000 | 0.000 | 0.000 | 0.000 | 0.000 |
| *Verrucosispora* | 0 | 25.00 | 4.70 | 9.60 | 54.62 | 40.0 | 0.000 | 0.000 | 0.000 | 0.000 | 0.000 |
| *Agrococcus* | 0 | 27.00 | 9.00 | 7.90 | 49.01 | 40.0 | 0.000 | 0.000 | 0.000 | 0.000 | 0.000 |
| *Alcaligenes* | 0 | 27.00 | 9.00 | 7.90 | 49.01 | 40.0 | 0.000 | 0.000 | 0.000 | 0.000 | 0.000 |
| *Arthrobacter* | 1 | 27.00 | 9.00 | 7.90 | 49.01 | 40.0 | 0.000 | 0.000 | 0.000 | 0.000 | 0.000 |
| *Azospirillum* | 0 | 27.00 | 9.00 | 7.90 | 49.01 | 40.0 | 0.000 | 0.000 | 0.000 | 0.000 | 0.000 |
| *Bacillus* | 0 | 27.00 | 9.00 | 7.90 | 49.01 | 40.0 | 0.000 | 0.000 | 0.000 | 0.000 | 0.000 |
| *Bradyrhizobium* | 1 | 27.00 | 9.00 | 7.90 | 49.01 | 40.0 | 0.000 | 0.000 | 0.000 | 0.000 | 0.000 |
| *Clavibacter* | 0 | 27.00 | 9.00 | 7.90 | 49.01 | 40.0 | 0.000 | 0.000 | 0.000 | 0.000 | 0.000 |
| *Deinococcus* | 0 | 27.00 | 9.00 | 7.90 | 49.01 | 40.0 | 0.000 | 0.000 | 0.000 | 0.000 | 0.000 |
| *Dermabacter* | 0 | 27.00 | 9.00 | 7.90 | 49.01 | 40.0 | 0.000 | 0.000 | 0.000 | 0.000 | 0.000 |
| *Detolaasinbacter* | 1 | 27.00 | 9.00 | 7.90 | 49.01 | 40.0 | 0.000 | 0.000 | 0.000 | 0.000 | 0.000 |
| *Janibacter* | 0 | 27.00 | 9.00 | 7.90 | 49.01 | 40.0 | 0.000 | 0.000 | 0.000 | 0.000 | 0.000 |
| *Microbacterium* | 0 | 27.00 | 9.00 | 7.90 | 49.01 | 40.0 | 0.000 | 0.000 | 0.000 | 0.000 | 0.000 |
| *Micrococcus* | 0 | 27.00 | 9.00 | 7.90 | 49.01 | 40.0 | 0.000 | 0.000 | 0.000 | 0.000 | 0.000 |
| *Mycobacterium* | 0 | 27.00 | 9.00 | 7.90 | 49.01 | 40.0 | 0.000 | 0.000 | 0.000 | 0.000 | 0.000 |
| *Nocardia* | 0 | 27.00 | 9.00 | 7.90 | 49.01 | 40.0 | 0.000 | 0.000 | 0.000 | 0.000 | 0.000 |
| *Nocardioides* | 0 | 27.00 | 9.00 | 7.90 | 49.01 | 40.0 | 0.000 | 0.000 | 0.000 | 0.000 | 0.000 |
| *proteobacteria* | 1 | 27.00 | 9.00 | 7.90 | 49.01 | 40.0 | 0.000 | 0.000 | 0.000 | 0.000 | 0.000 |
| *Pseudomonas* | 0 | 27.00 | 9.00 | 7.90 | 49.01 | 40.0 | 0.000 | 0.000 | 0.000 | 0.000 | 0.000 |
| *Rhodococcus* | 1 | 27.00 | 9.00 | 7.90 | 49.01 | 40.0 | 0.000 | 0.000 | 0.000 | 0.000 | 0.000 |
| *Sphingomonas* | 0 | 27.00 | 9.00 | 7.90 | 49.01 | 40.0 | 0.000 | 0.000 | 0.000 | 0.000 | 0.000 |
| *Staphylococcus* | 0 | 27.00 | 9.00 | 7.90 | 49.01 | 40.0 | 0.000 | 0.000 | 0.000 | 0.000 | 0.000 |
| *Stenotrophomonas* | 1 | 27.00 | 9.00 | 7.90 | 49.01 | 40.0 | 0.000 | 0.000 | 0.000 | 0.000 | 0.000 |
| *Streptomyces* | 1 | 27.00 | 9.00 | 7.90 | 49.01 | 40.0 | 0.000 | 0.000 | 0.000 | 0.000 | 0.000 |
| *Terrabacter* | 0 | 27.00 | 9.00 | 7.90 | 49.01 | 40.0 | 0.000 | 0.000 | 0.000 | 0.000 | 0.000 |
| *Variovorax* | 0 | 27.00 | 9.00 | 7.90 | 49.01 | 40.0 | 0.000 | 0.000 | 0.000 | 0.000 | 0.000 |
| *Verrucosispora* | 0 | 27.00 | 9.00 | 7.90 | 49.01 | 40.0 | 0.000 | 0.000 | 0.000 | 0.000 | 0.000 |

Data Set Two: Fungi in Chernobyl Reactor Buildings

This data set was generated by mycological analysis of 24 samples collected within the Chernobyl nuclear power plant buildings11-12 years after the accident, presented in Table 1 of reference [2].

| **Species** | **Number of low-dose-rate samples in which species was detected (out of 15)** | **Number of high-dose-rate samples in which species was detected (out of 9)** |
| --- | --- | --- |
| *Cladosporium cladosporioides* | 4 | 1 |
| *C. herbarum* | 3 | 4 |
| *C. sphaerospermum* | 11 | 9 |
| *Cladosporium sp.* | 1 | 1 |
| *Phialophora melinii* | 0 | 2 |
| *Aureobasidium pullulans* | 3 | 4 |
| *A. versicolor* | 4 | 5 |
| *Sydowia polyspora* | 1 | 0 |
| *Aspergillus flavus* | 0 | 1 |
| *A. fresenii* | 0 | 1 |
| *A. fumigatus* | 1 | 1 |
| *A. niger* | 2 | 2 |
| *A. ochraceus* | 0 | 1 |
| *A. ustus* | 0 | 1 |
| *Paecilomyces variotii* | 0 | 1 |
| *Penicillium chrysogenum* | 1 | 0 |
| *P. citrinum* | 0 | 1 |
| *P. hirsutum* | 1 | 8 |
| *P. hordei* | 1 | 0 |
| *P. ingelheimense* | 2 | 0 |
| *Doratomyces stemonitis* | 1 | 1 |
| *Chaetomium globosum* | 3 | 2 |
| *Botrytis cinerea* | 2 | 1 |
| *Acremonium strictum* | 5 | 2 |
| *Beauveria bassiana* | 0 | 1 |
| *Fusarium merismoides* | 1 | 0 |
| *F. oxysporum* | 1 | 1 |
| *F. solani* | 2 | 1 |
| *Stachybotrys chartarum* | 1 | 1 |
| *Chrysosporium pannorum* | 1 | 0 |
| *Alternaria alternata* | 6 | 4 |
| *Ulocladium botrytis* | 1 | 0 |
| *orange sterile mycelium* | 2 | 0 |
| *white sterile mycelium* | 1 | 2 |
| *Geotrichum candidum* | 0 | 1 |
| *Geotrichum sp.* | 1 | 0 |
| *Mucor plumbeus* | 1 | 0 |

Data Set Three: Continuously Irradiated Yeast

This data set was produced by continuous ^60^Co γ-irradiation of diploid yeast (*Saccharomyces cerevisiae*, strain 211) in a laboratory chemostat for multiple generations [3]. It was presented in Tables 1-2 of reference [3]. The value 0* indicates that the corresponding dose rate was the critical dose rate for population extinction.

| **Dilution rate (h^-1^)** | **Dose rate (Gy/h)** | **Equilibrium cell**  **concentration**  **(10^6^ cells/ml)** |
| --- | --- | --- |
| 0.06 | 0 | 480 |
| 0.1 | 0 | 310 |
| 0.2 | 0 | 300 |
| 0.25 | 0 | 270 |
| 0.3 | 0 | 220 |
| 0.06 | 45.0 | 65 |
| 0.1 | 45.0 | 60 |
| 0.2 | 30.0 | 77 |
| 0.25 | 24.0 | 52 |
| 0.3 | 13.8 | 86 |
| 0.06 | 180.0 | 0* |
| 0.1 | 180.0 | 0* |
| 0.2 | 45.0 | 0* |
| 0.25 | 45.0 | 0* |
| 0.3 | 24.0 | 0* |

**References**

1. Fredrickson JK, Zachara JM, Balkwill DL, Kennedy D, Shu-mei WL, Kostandarithes HM, et al. Geomicrobiology of high-level nuclear waste-contaminated vadose sediments at the Hanford Site, Washington State. Appl Environ Microb. 2004;70(7):4230-41.

2. Zhdanova NN, Zakharchenko VA, Vember VV, Nakonechnaya LT. Fungi from Chernobyl: mycobiota of the inner regions of the containment structures of the damaged nuclear reactor. Mycological research. 2000;104(12):1421-6.

3. Kiefer J, Al-Talibi AA, Doll G. Radiosensitivity of continuous cultures. II. Continuous gamma-ray exposure. Radiation research. 1977;69(2):230-40. PubMed PMID: 841001.
