# Supplementary material for: Quantitative Modeling of Microbial Population Responses to Chronic Irradiation Combined with Other Stressors
Source: PLoS One. 2016 Jan 25;11(1):e0147696. doi: 10.1371/journal.pone.0147696 (PMC4726741; doi:10.1371/journal.pone.0147696)
Supplement: S2 Appendix — (DOCX) [file pone.0147696.s002.docx]

**S2 Appendix: Analysis of Data Set One: Bacteria at the Hanford Nuclear Waste Site**

*Filter Procedure for Identifying Important Predictor Variables*

The goal of this procedure was to identify the most important variables for describing the data, and then to construct GLMMs using these variables. We used machine learning and information theoretic approaches. Machine learning algorithms are evolving rapidly in recent years due to the growing power of computers available to researchers. Compared with more traditional modeling approaches, these algorithms often exhibit greater power for explaining and predicting complex dependences in data sets which contain multiple potential predictor variables, as often occurs in ecology [1-3]. Information theoretic methods, which represent extensions of likelihood theory, are also very useful for the analysis of complex data from observational ecological studies [4, 5]. In particular, these methods allow not only the selection of a single best-fitting model, but provide an easily-implementable approach for making inferences from several models weighted by the strength of evidence supporting each model [4, 5].

The filter procedure consisted of the following steps:

Step 1: Because the contaminated soil samples were collected from different depths below the ruptured nuclear waste tank, it would be plausible to hypothesize that these samples are statistically dependent: e.g. it would be more likely that samples separated by 1 meter of soil depth would contain the same bacterial taxa, than samples separated by 10 meters. We used Mantel tests for spatial auto-correlation (using the *ade4* *R* package) to quantitatively test this hypothesis.

Step 2: To screen out those variables which are least important for describing the data and are likely to be associated with the outcome merely by chance, we used two machine learning ensemble prediction methods: random forests (RF) [6] and random generalized linear modeling (RGLM) [7]. These methods were implemented in the *randomForest.ddR* and *randomGLM* *R* packages, respectively. Both RF and RGLM were used on the same data to increase robustness of the findings: those variables that are identified as unimportant by both machine learning methods are most likely to be noise variables.

Briefly, RF is a decision tree-based machine learning method for classification and regression, which relies on bootstrap aggregation (“bagging”) [6]. A tree is trained on a given bootstrapped sample, and its performance is tested on data which was not included in the training sample. This procedure is repeated multiple times. The relative importance of different predictor variables is assessed by the variable importance metric (VIM) – e.g. the difference in the RF error rates which can be attributed to the given variable based on random permutation of variables.

RGLM is a newer ensemble prediction method based on generalized linear models (GLMs) whose covariates are selected using forward regression according to the Akaike information criterion (AIC) [4, 5]. RGLM showed similar or superior prediction accuracy compared with RF and some other methods on several simulated and machine learning benchmark data sets [7]. In RGLM, VIM can be estimated by the number of times when the given variable is selected by forward regression across all bags. For example, a variable which was selected in 50 out of 100 bags is considered more important for describing the data, than a variable which was selected in only 5 bags.

To obtain robust results, we sought to establish the threshold for variable importance (VIM_crit_), which would be intended to separate biologically-meaningful variables from noise variables. In other words, for each machine learning method we estimated VIM_crit_, such that all variables with VIM > VIM_crit_ are likely to be meaningful predictors, whereas those with VIM < VIM_crit_ are likely to be noise. Only those variables with VIM > VIM_crit_ for either RF or RGLM were retained for further analysis, and all others were discarded.

To estimate VIM_crit_ for the data set analyzed here, we generated simulated “noise” variables by drawing random numbers from the normal, log-normal, binomial, exponential, and uniform distributions – three variables from each distribution. In addition, we created three variables from the equation exp[RN×sin(depth/5)], where RN is a uniformly-distributed random number (between zero and 1), and depth is the depth below the soil surface in meters. Therefore, 18 noise variables were produced, the first 15 of which were completely random and the last 3 had an oscillating dependence on depth. These noise variables were normalized so that their medians equaled the median of the 10 observed variables (depth, water content, conductivity, temperature, pH, ^137^Cs, ^99^Tc, Cr, NO_2_, and NO_3_) – this was done so that all variables would be on an approximately equal scale. The noise variables were added to the observed data set, and the combined observed and simulated data were analyzed by the machine learning methods.

For RF, it has been shown that reliability of results is increased by: (1) limiting the terminal node size of the decision trees (e.g. to 10% of the sample size), and (2) repeating the analysis several times with different initial random seeds, so that only those variables with consistently high VIMs across several repeats are likely to be meaningful [3]. Therefore, we analyzed the data set (which contained 10 observed variables and 18 simulated noise variables) ten times using RF in regression mode. We calculated the median absolute value of the VIMs for all 18 noise variables. Then we calculated the relative importance score (RIS) [3] for each of the 10 observed variables by dividing the variable's VIM by the median VIM for noise variables in the given RF run. Next, we calculated the median RIS for each variable (RISMED) across all 10 RF runs. Then we calculated the maximum of the median RIS values for all noise variables (RISMAX_noise_). Finally, we compared RISMED with RISMAX_noise_ and discarded from further analysis all variables with RISMED < RISMAX_noise_.

In other words, for the RF method, VIM_crit_ was set equal to RISMAX_noise_. All variables whose median RIS across 10 independent RF runs was smaller than the maximum of the median RIS values for random noise variables were considered likely to be noise. This procedure was intended to retain for further analysis only those variables with robust main effects, which recurred in several RF runs.

For the RGLM method, where VIM was defined differently than in RF, VIM_crit_ was also estimated by a different procedure described as follows. The data set was analyzed by RGLM ten times with different initial random seeds. In each run, the number of bags in which a given variable was selected by forward regression (using AIC) was defined as the variable’s VIM. Only those variables for which VIM was greater than a threshold value (NBAGS_crit_) were considered important in the given RGLM run. We tried several values of NBAGS_crit_ (e.g. 30, 50, 100, 150, 200, and 250 out of 300 bags) to asses at which value the "signal/noise ratio" (R_sig_) would be maximized. R_sig_ was defined as the ratio SUM_sig_/SUM_noise_, where SUM_sig_ is the sum of the numbers of RGLM runs (out of 10) when any of the 10 observed variables were selected as important at the given NBAGS_crit_ value, and SUM_noise_ is the corresponding value for noise variables.

For example, suppose that at a certain NBAGS_crit_ value 4 observed variables are selected in all 10 RGLM runs and one additional observed variable is selected in 6 runs. Then SUM_sig_ would be 4×10+1×6=46. Now suppose that 1 noise variable was selected in 7 RGLM runs and two other noise variables in 3 runs. Then SUM_noise_ would be 1×7+2×3=13, and R_sig_ = 46/13 = 3.54. This calculation was performed for all tried values of NBAGS_crit_, and the value of NBAGS_crit_ at which R_sig_ was maximized was selected as VIM_crit_ for the RGLM method.

Consequently, for the RF and RGLM methods we used random noise variables as “benchmarks” to separate those variables with potentially meaningful main effects from those variables which are likely to unimportant for describing the data. We discarded from further analysis all of the latter variables (i.e. those with VIM < VIM_crit_) for both RF and RGLM methods.

To validate this procedure for selecting important variables, we tested it as follows. We created an *a priori* defined “true” logistic model using observed values of water content, temperature, conductivity, ^137^Cs, Cr, and NO_2_ as predictors. A uniformly-distributed random number was also added to the model as the intercept. The model was used to simulate presence/absence of bacterial taxa in different samples: all predicted probabilities >0.5 were converted to presence (i.e. values of 1), and those <0.5 were converted to absence (i.e. values of 0). The parameters for this true model were chosen so that the simulated outcome resembled the observed data – e.g. contained approximately the same number of presence values as the real data. The resulting data set which consisted of observed values of all variables and the previously created random noise variables, together with a true model-simulated outcome, was analyzed 10 times by RF and RGLM methods as described above.

The goal of this procedure was to evaluate the performances of RF and RGLM in: (1) assigning consistently high VIMs to those variables (water content, temperature, conductivity, ^137^Cs, Cr, and NO_2_) which were used in the true model, and (2) assigning consistently low VIMs to the other variables, which were not used in the true model. The results were fairly strong: across repeated runs using different initial random seeds, the RF and RGLM methods with VIM_crit_ thresholds estimated as described above assigned VIM > VIM_crit_ to 4-6 out of the 6 variables used in the true model, and to only 1-3 variables not used in the model. In other words, the majority (57-100%) of variables designated as important were indeed important, i.e. present in the true model.

Step 3: Those variables which were retained as potentially important in step 2 were used to construct a logistic model. As mentioned in the main text, the distributions of radionuclides and chemical contaminants released from a common source (the ruptured nuclear waste tank) were correlated. Consequently, multi-collinearity of variables was a potentially important problem. We assessed it by calculating the variance inflation factor (VIF) for each predictor. We sequentially removed variables with the highest VIFs from the model, until all variables had VIF < 2.

Step 4: The set of variables retained after step 3 was called the confidence set of fixed-effect models. It was exhaustively searched using the *glmulti* routine in the *MuMin R* package. All possible combinations of these variables were screened, and support for each combination was assessed by the sample-size corrected Akaike information criterion (AICc) [4, 5]. Using multi-model inference (MMI), all variables were ranked in order of importance, where the importance of a given variable was the sum of Akaike weights of all models which contained this variable [4]. Model-averaged regression coefficients (and their uncertainties) were also produced by MMI [4].

Step 5: The variable combination from step 4 which produced the lowest AICc score, indicating the highest support from the data, was called the best-supported fixed-effect model. We performed regression diagnostics on this model as follows: (1) possible outlier data points were detected using the Cook's distance threshold of 4/(S – κ – 1), where S is sample size and κ is number of predictors, and the effects of these outliers were assessed by robust logistic regression (the *glmRob* routine in R); (2) potential overdispersion of the data was assessed by fitting logistic models with binomial vs. quasi-binomial error distributions and comparing them by the X^2^ test; (3) potential autocorrelation of residuals was assessed by the Durbin Watson test.

Step 6: Predictive performance of the best-supported fixed-effect model was evaluated using the area under the ROC curve [8] and by K-fold cross-validation [9].

Step 7: Lastly, we considered random effects (both intercepts and slopes) for each variable present within the best-supported fixed-effect model. AICc is not reliable for mixed-effect models because there is no clear-cut approach for quantifying the number of parameters for the random effects [10]. Consequently, we added random effects for one variable at a time and compared the performances of the resulting mixed-effect models by absolute GOF quantified by the *r.squaredGLMM* function from the *MuMIn R* package. This function calculates marginal and conditional R^2^ [11, 12]. Based on this approach, we interpreted the mixed-effect model which generated the largest conditional R^2^ (using fixed effects for variables represented within the best-supported fixed-effect model, plus random effects for one of these variables) as the as best-fitting mixed-effect model.

*Assumptions Made During Data Analysis*

The GLMM-based methods described in the main text relate the probability to detect a given taxon in a given sample to conditions within the sample (e.g. concentrations of radionuclides and chemical contaminants) in a simple manner - using a linear dependence with a logit link. They also allow responses of different taxa to the same stressor to be different - using normally-distributed random effects. However, interactions between taxa are not accounted for.

Due to the large number (26) and diversity of taxa [13], we had insufficient information about interactions between taxa, and had no choice but to ignore them. However, because the GLMM approach turned out to describe the data not too badly (presented in Results section of the main text), interactions between taxa were probably not dominant over the effects of the available predictor variables (radionuclides, chemical contaminants).

Due to potential multi-collinearity of variables, we did not explore quadratic terms and multi-variable interactions. Consequently, it is conceivable that certain important effects were not found. For example, two distinct variables with seemingly negligible effects when considered one at the time can still yield a dramatic effect whenever they are combined [3]. We believe that such situations are possible, but not likely in this data set because there are no mechanistic arguments for why any of the potential predictors would not have an effect alone, but only in combination with others. For example, the effect of radiation from ^137^Cs may be modulated by chemical toxins, but should be discernible even in the absence of these toxins. Moreover, some potential interaction terms (e.g. depth×pH) do not make sense mechanistically.

Consequently, despite these potential drawbacks, we believe that the use of GLMMs and the filter procedure made the data analysis focused and computationally feasible, and was better-justified than alternative approaches for analyzing the selected data. For instance, an alternative method where the number of taxa detected in each sample would be modeled assuming Poisson-distributed errors would be less informative because not only interactions between taxa, but also taxon-specific differences in responses would not be included (i.e. all taxa would be assumed to respond identically to a given stressor).

**References**

1. Olden JD, Lawler JJ, Poff NL. Machine learning methods without tears: a primer for ecologists. The Quarterly review of biology. 2008;83(2):171-93.

2. Singal AG, Mukherjee A, Elmunzer BJ, Higgins PD, Lok AS, Zhu J, et al. Machine learning algorithms outperform conventional regression models in predicting development of hepatocellular carcinoma. The American journal of gastroenterology. 2013;108(11):1723-30.

3. Holzinger ER, Szymczak S, Dasgupta A, Malley J, Li Q, Bailey-Wilson JE. Variable selection method for the identification of epistatic models. Pac Symp Biocomput. 2015:195-206. PubMed PMID: 25592581; PubMed Central PMCID: PMCPMC4299919.

4. Burnham KP, Anderson DR. Model selection and multi-model inference: a practical information-theoretic approach: Springer; 2002.

5. Burnham KP, Anderson DR, Huyvaert KP. AIC model selection and multimodel inference in behavioral ecology: some background, observations, and comparisons. Behav Ecol Sociobiol. 2011;65(1):23-35. doi: DOI 10.1007/s00265-010-1029-6. PubMed PMID: WOS:000285786000003.

6. Breiman L. Random forests. Machine learning. 2001;45(1):5-32.

7. Song L, Langfelder P, Horvath S. Random generalized linear model: a highly accurate and interpretable ensemble predictor. BMC bioinformatics. 2013;14(1):5.

8. Hanley JA, McNeil BJ. The meaning and use of the area under a receiver operating characteristic (ROC) curve. Radiology. 1982;143(1):29-36.

9. Kohavi R, editor A study of cross-validation and bootstrap for accuracy estimation and model selection. Ijcai; 1995.

10. Grueber C, Nakagawa S, Laws R, Jamieson I. Multimodel inference in ecology and evolution: challenges and solutions. Journal of evolutionary biology. 2011;24(4):699-711.

11. Nakagawa S, Schielzeth H. A general and simple method for obtaining R2 from generalized linear mixed‐effects models. Methods in Ecology and Evolution. 2013;4(2):133-42.

12. Johnson PC. Extension of Nakagawa & Schielzeth's R2GLMM to random slopes models. Methods in Ecology and Evolution. 2014;5(9):944-6.

13. Fredrickson JK, Zachara JM, Balkwill DL, Kennedy D, Shu-mei WL, Kostandarithes HM, et al. Geomicrobiology of high-level nuclear waste-contaminated vadose sediments at the Hanford Site, Washington State. Appl Environ Microb. 2004;70(7):4230-41.
