# Supplementary material for: Quantitative Modeling of Microbial Population Responses to Chronic Irradiation Combined with Other Stressors
Source: PLoS One. 2016 Jan 25;11(1):e0147696. doi: 10.1371/journal.pone.0147696 (PMC4726741; doi:10.1371/journal.pone.0147696)
Supplement: S4 Appendix — (DOCX) [file pone.0147696.s004.docx]

**S4 Appendix: Worked Example of Mechanistic Modeling Approach Used for Analysis of Data Set Three: Continuously Irradiated Yeast**

*Mechanistic Mathematical Models*

The basic mechanistic formalism (model M_1_, Table 2 of the main text) has the following structure, where N is the yeast cell concentration, *d* is the chemostat dilution rate, R is radiation dose rate, and *m*, *g*, *q*, δ, and *k* are parameters:

*d*N/*dt*  = (–*d* – *m*×N + *g* + *q*×exp(–δ×R) – *k*×R )×N (S4)

This formalism contains the following assumptions: Radiation-induced cell killing is proportional to dose rate. It is represented by –*k*×R. Radiation effects on cell proliferation are non-linear. The term *q*×exp(–δ×R) represents the proliferation rate component (parameter *q*) which is suppressed by radiation (through parameter δ). However, there is also another proliferation rate component (parameter *g*) which is not affected by radiation. The decrease in net cell proliferation rate with increasing cell concentration is described by –*m*×N, where *m* is the coefficient of intraspecific competition. Chemostat dilution rate (*d*) also reduces the net cell proliferation rate.

The equilibrium cell concentration (N_eq_) could be attained if the dose rate and/or dilution rate were sufficiently low [1]. At equilibrium *d*N/*dt* should equal zero, producing the following equation, where N_eq(M)_ is the equilibrium cell concentration predicted by model M_1_:

0 = (–*d* – *m*×N_eq(M)_ + *g* + *q*×exp(–δ×R) – *k*×R )×N_eq(M)_ (S5)

The solution of Eq. S5 for N_eq(M)_ is:

N_eq(M)_ = (–*d* + *g* + *q*×exp(–δ×R) – *k*×R)/*m* (S6)

Eq. S6 shows that predicted N_eq(M)_ is positive only if *g* + *q*×exp(–δ×R) > *d* + *k*×R, i.e. only if the cell proliferation rate (which is affected by radiation) is larger than the sum of cell removal rates caused by dilution and radiation. If the dose rate and/or dilution rate become too high, predicted N_eq(M)_ will become negative, i.e. the population will go extinct.

The critical dose rate (R_crit(M)_), above which population extinction is predicted, can be found by substituting N_eq(M)_ = 0 into Eq. S6 and solving the resulting equation for R_crit(M)_:

0 = (–*d* + *g* + *q*×exp(–δ×R_crit(M)_) – *k*×R_crit(M)_)/*m* (S7)

The solution can be calculated using the Lambert *LW* function:

$R_{crit(M)}=\frac{1}{k\times\delta}\left( k\times LW\left( \delta\times\frac{q}{k}\times exp\left( \left( d - g \right)\times\frac{\delta}{k} \right) \right)-\delta\times\left( d - g \right) \right)$ (S8)

To explore other biologically-plausible possibilities for how radiation and/or dilution rate can affect the yeast population, we generated additional model versions (M_2_-M_20_, summarized in Table 2 of the main text). For each model, we calculated solutions for N_eq_ and R_crit_, following the same steps described above for model M_1_. To describe more complex system behaviors than in model M_1_, in some alternative formalisms we introduced extra parameters called μ and σ.

*Model Parameters*

Because the analyzed data set was quite small, we reduced the number of adjustable model parameters as much as possible. The experimenters in [1] reported that, even at very low dilution rates, extinction occurred if the radiation dose rate was above a limiting value R_L_ (about 300 Gy/h). We used this information to analytically solve for parameter *g*, thereby reducing the number of adjustable parameters by one. For model M_1_, this calculation involved substituting *d* = 0 and R_crit(A)_ = R_L_ into Eq. S8, producing the following equation:

$R_{L}=\frac{1}{k\times\delta}\left( k\times LW\left( \delta\times\frac{q}{k}\times exp\left( -g\times\frac{\delta}{k} \right) \right)+\delta\times g \right)$ (S9)

Solving Eq. S9 for parameter *g* produced the following result:

*g* = *k*×R_L_– *q*×exp(–δ×R_L_) (S10)

Because *g* was solved for analytically, only four adjustable parameters (*m*, *q*, δ, *k*) remained in model M_1_. The same steps were implemented for all alternative models which contained parameter *g* (all formalisms except M_11_ and M_18_).

*Model Fitting Procedure*

All models (M_1_-M_20_) were fitted to data by maximizing the log likelihood, using optimization routines in Maple 17® software. The probability of finding the global (rather than local) maximum was enhanced by using 100 random initial conditions for the adjustable parameters. All parameters were restricted to ≥ 0 to maintain mechanistic plausibility.

We assumed Gaussian errors with magnitudes proportional to the mean values. This assumption was reasonable because the equilibrium cell concentrations and critical dose rates were continuous variables, and the errors introduced during measurement of these variables were not reported by the experimenters [1] but were likely to be proportional to the data point values. Moreover, this assumption allowed each model to be fitted to the two types of data simultaneously using a combined expression for the log likelihood, even though the two data types have different magnitudes and units.

The log likelihood function (LL_(x)_) has the following structure, where X is the model (any of the formalisms described in Table 2 of the main text), _p_N_eq(x,_*_i_*_)_ is the predicted equilibrium cell concentration for the *i*-th data point, _o_N_eq(_*_i_*_)_ is the corresponding observed value, _p_R_crit(x,_*_j_*_)_ is the predicted critical dose rate for the *j*-th data point, _o_R_crit(_*_j_*_)_ is the corresponding observed value, ν is the total number of data points for equilibrium cell concentration, and ζ is the number of data points for critical dose rate:

LL_(x )_= – ½(ν + ζ)×(ln[SS/(ν + ζ)] + ln[2π] + 1),

where SS = ∑*_i_*[(_p_N_eq(x,_*_i_*_)_ – _o_N_eq(_*_i_*_)_)^2^/_o_N_eq(_*_i_*_)_^2^] + ∑*_j_*[(_p_R_crit(x,_*_j_*_)_ – _o_R_crit(_*_j_*_)_)^2^/_o_R_crit(_*_j_*_)_^2^] (S11)

The constant term ln[2π] + 1 was included for completeness, but had no effect on the comparison of model performances and on parameter estimation.

Absolute GOF for the analyzed models was assessed by exploratory data fitting using visual inspection of model fits and linear regression of model predictions vs. observed data points. These approaches showed that several tested models performed reasonably: model prediction curves visually passed close to the data points, and the 95% confidence intervals (CIs) for the regression of model predictions vs. data included 0 for the intercept and 1 for the slope.

*Estimation of Model Parameter Uncertainties*

Because the magnitude of errors in the experimental data was unknown [1], we explored the effects of random perturbations of the data on best-fit model parameter values by Monte Carlo simulation. One thousand random synthetic data sets were produced from the original data set by replacing the observed data point values with random numbers drawn from the Normal distribution, where the mean was set to the observed data point value and the standard deviation was set to ¼ of the observed data point value. This assumed standard deviation probably overestimated the experimental errors, but was useful for assessing the robustness of model predictions and parameter values against large random fluctuations of the data. The model of interest was then fitted to each random data set, and the best-fit parameter values and model predictions for each data point were recorded. The 2.5-th and 97.5-th percentiles of the distribution of values for each parameter were used to estimate the 95% confidence intervals, and the same method was applied to model predictions.

*Sensitivity of Model Predictions to Parameter Values*

To identify which parameters are most influential for model predictions, we performed sensitivity analysis. A selected parameter was increased by 10% above its best-fit value while holding all other parameters constant at their best-fit values and the resulting model prediction was recorded. This procedure was repeated sequentially for each parameter.

*MMI Predictions*

All highly supported models (with ΔAICc < 6) were used to generate MMI predictions for the critical radiation dose rate (R_crit_) above which the yeast population becomes extinct.

*Assumptions Made During Data Analysis*

We believe that mechanistic mathematical models provide important advantages over purely descriptive approaches [2, 3]. In particular, mechanistic models provide explanations for the processes which produced the data, whereas descriptive models seek merely to describe statistically the data. Consequently, a mechanistic model, which includes the most important processes affecting the studied system, is more likely than a descriptive model to produce useful predictions outside the data set on which it was initially calibrated [3].

In contrast to the previous two data sets, which contain multiple predictor variables and/or taxa and insufficient information about inter-taxon interactions, the current data set involved only one taxon (*S. cerevisiae*) and two predictor variables (radiation and chemostat dilution), and was therefore amenable to mechanistic analysis. We constructed mechanistic formalisms (described above) which modeled the rate of change of the cell concentration over time using differential equations. The main processes involved were assumed to be cell proliferation, death and intraspecific competition. Radiation and/or dilution rate, possibly interacting with each other, were assumed to influence some or all of these processes.

**References**

1. Kiefer J, Al-Talibi AA, Doll G. Radiosensitivity of continuous cultures. II. Continuous gamma-ray exposure. Radiation research. 1977;69(2):230-40. PubMed PMID: 841001.

2. Bolker BM. Ecological models and data in R: Princeton University Press; 2008.

3. Hilborn R, Mangel M. The ecological detective: confronting models with data: Princeton University Press; 1997.
